# Brain Age Prediction: A Comparison between Machine Learning Models Using Brain Morphometric Data

**DOI:** 10.3390/s22208077

**Published:** 2022-10-21

**Authors:** Juhyuk Han, Seo Yeong Kim, Junhyeok Lee, Won Hee Lee

**Affiliations:** Department of Software Convergence, Kyung Hee University, Yongin 17104, Korea

**Keywords:** brain age prediction, structural magnetic resonance imaging, machine learning, brain morphometry

## Abstract

Brain structural morphology varies over the aging trajectory, and the prediction of a person’s age using brain morphological features can help the detection of an abnormal aging process. Neuroimaging-based brain age is widely used to quantify an individual’s brain health as deviation from a normative brain aging trajectory. Machine learning approaches are expanding the potential for accurate brain age prediction but are challenging due to the great variety of machine learning algorithms. Here, we aimed to compare the performance of the machine learning models used to estimate brain age using brain morphological measures derived from structural magnetic resonance imaging scans. We evaluated 27 machine learning models, applied to three independent datasets from the Human Connectome Project (HCP, *n* = 1113, age range 22–37), the Cambridge Centre for Ageing and Neuroscience (Cam-CAN, *n* = 601, age range 18–88), and the Information eXtraction from Images (IXI, *n* = 567, age range 19–86). Performance was assessed within each sample using cross-validation and an unseen test set. The models achieved mean absolute errors of 2.75–3.12, 7.08–10.50, and 8.04–9.86 years, as well as Pearson’s correlation coefficients of 0.11–0.42, 0.64–0.85, and 0.63–0.79 between predicted brain age and chronological age for the HCP, Cam-CAN, and IXI samples, respectively. We found a substantial difference in performance between models trained on the same data type, indicating that the choice of model yields considerable variation in brain-predicted age. Furthermore, in three datasets, regularized linear regression algorithms achieved similar performance to nonlinear and ensemble algorithms. Our results suggest that regularized linear algorithms are as effective as nonlinear and ensemble algorithms for brain age prediction, while significantly reducing computational costs. Our findings can serve as a starting point and quantitative reference for future efforts at improving brain age prediction using machine learning models applied to brain morphometric data.

## 1. Introduction

Neuroimaging-based brain age is widely used as a biomarker to quantify the progress of brain diseases and aging [1]. The biological age of the brain (“brain age”) is estimated typically by applying a machine learning approach to magnetic resonance imaging (MRI) data to predict chronological age. The difference between an individual’s predicted brain age and actual chronological age is referred to here as brain-predicted age difference (brainPAD) [2,3], which is also known as brain age gap [4,5] or brain age delta [6]. This metric reflects the deviation from expected age trajectories and is often used to index brain health [1]. A positive brainPAD indicates that an individual’s brain age is higher than their actual age, which is referred to as accelerated aging [5]. A negative brainPAD reflects a lower brain-predicted age, referred to as delayed aging [5]. This empirical measure of brainPAD derived from the general population has proven to be a useful marker of neurodegeneration and cognitive decline in clinical populations [7,8,9,10]. Elevated brain age relative to chronological age has been associated with lower cognitive capacity, well-being and general health [11], adverse physical [8], and mental health phenotypes [2,12]. Collectively, these studies provide evidence to support the use of brain-predicted age as a biomarker for brain health.

A number of machine learning studies have been conducted to predict brain age, most commonly based on structural MRI data [2,13,14,15,16]. Brain morphological features extracted from structural MRI scans have been widely used, since they allow the morphological age-related brain changes to be examined in a great variety of disorders and conditions [17,18,19,20,21]. The literature shows great variability in methods, including the choice of machine learning algorithms and their parameters, sample size, sample composition, and the type of input features [5,13]. Many different machine learning approaches exist for brain age prediction. Typically, a single machine learning algorithm, such as Support Vector Regression (SVR), Relevance Vector Regression (RVR), and Gaussian Process Regression (GPR), has been commonly used for brain age prediction [8,14,15,22,23]. Previous studies have compared different machine learning models applied to the same data [2,14,15,16]. However, there is a significant gap regarding the accuracy of various machine learning algorithms in brain age prediction, and comparative performance of different machine learning algorithms has not been comprehensively evaluated.

Here, we focus exclusively on the evaluation of various machine learning algorithms used to predict brain age using brain morphological features derived from structural MRI data. To this end, we used publicly available samples of healthy individuals from the Human Connectome Project (HCP), the Cambridge Centre for Ageing and Neuroscience (Cam-CAN), and the Information eXtraction from Images (IXI). Three independent datasets were used to test the robustness of the results to sample composition. We evaluated 27 machine learning algorithms applied to the same morphometric data and then assessed their performance in a hold-out test set within each sample. The algorithms tested included parametric and nonparametric, linear and nonlinear, and Bayesian, kernel-based and tree-based models. This study aims at providing a guide for choosing the appropriate machine learning models when predicting brain age based on brain morphometric data, due to its enormous benefits in age-related disorders.

## 2. Materials and Methods

### 2.1. Datasets

Three independent datasets were considered: the Human Connectome Project (HCP) S1200 release (*n* = 1113, 606 females, age range 22–37 years) [24], the Cambridge Centre for Ageing and Neuroscience (Cam-CAN) (*n* = 601, 302 females, age range 18–88 years) [25], and the Information eXtraction from Images (IXI) (*n* = 567, 316 females, age range 19–86 years) (https://brain-development.org, accessed on 1 September 2020). All individuals were screened according to local study protocols to ensure they had no history of neurological, psychiatric, or major medical conditions. T1-weighted MRI scans were acquired at 1.5T or 3T scanners with standard T1-weighted MRI sequences. Details about the acquisition protocol and pipelines are described elsewhere for HCP [26], Cam-CAN [25], and IXI (https://brain-development.org, accessed on 1 September 2020). We used deidentified data from publicly available repositories. Ethical approvals and informed consents were obtained locally for each study, covering both participation and subsequent data sharing.

### 2.2. Image Processing and Feature Extraction

Structural T1-weighted images were processed as described previously [2]. The same preprocessing pipeline was applied in the three datasets to extract brain morphometric measures using FreeSurfer 6.0 (http://surfer.nmr.mgh.harvard.edu, accessed on 1 September 2020). Briefly, the cortical surface for each participant was reconstructed from their T1-weighted image by the following steps: skull stripping, segmentation of cortical gray and white matter, separation of the two hemispheres and subcortical structures, and construction of smooth representation of the gray/white matter boundary and the pial surface. Further technical details about the pipeline were described elsewhere [27,28].

Consistent with our previous study [2], the features were derived using the Desikan-Killiany cortical atlas [29], including global and region-specific measures of cortical thickness and surface area, in addition to the classic set of subcortical volume parcellation and summary statistics based on the automatic segmentation in FreeSurfer (Supplementary Figure S1). We chose to use the Desikan-Killiany parcellation as it is amongst the most widely used atlases in neuroimaging studies [30]. In each participant’s dataset, this procedure generated measures of total intracranial volume (ICV) and regional measures of cortical thickness (*n* = 68), surface area (*n* = 68), and subcortical volumes (*n* = 16) (for the complete list, see Supplementary Table S1). A feature matrix consisting of brain morphological measures (cortical thickness, surface area, subcortical volume, and total intracranial volume) was used for brain age prediction.

### 2.3. Machine Learning Algorithms

Brain age prediction was conducted using the Python machine learning framework PyCaret, which is an open-source, low-code machine learning library that automates machine learning workflow [31]. The PyCaret library was chosen because it requires significantly fewer lines of code to run various machine learning models and forms a pipeline consisting of all necessary blocks of functions or modules that can simplify the model training process. We employed 27 machine learning algorithms as described below.

#### 2.3.1. Parametric Algorithms

##### Linear Models

- Linear Regression (LR) [15,32]: this is an approach to fit a linear model by minimizing the residual sum of squares between the observed value and the value predicted by the ordinary least squares regression model.
- Least Absolute Shrinkage and Selection Operator (Lasso) Regression [2,33]: this is a linear algorithm that minimizes the residual sum of squares subject to the sum of the absolute value of the coefficients being less than a constant. This algorithm tends to produce some coefficients that are exactly zero.
- Ridge Regression [2,15,32]: this is a model tuning approach that is used to analyze the data that suffer from multi-collinearity. This method uses L2-norm regularization. When the issue of multi-collinearity occurs, least squares are unbiased and variance is significant. This algorithm shrinks the coefficients and it helps to reduce the model complexity and multi-collinearity.
- Elastic Net Regression [2,34]: this is a regularized linear regression model that combines both the L1 and L2 penalty functions. This algorithm performs variable selection and regularization simultaneously. This method is most appropriate where the number of features is greater than the number of samples. This allows the number of selected features to be larger than the sample size while achieving a sparse model.
- Least Angle Regression (LAR) [35]: this algorithm is similar to forward stepwise regression. It finds a variable that is most highly correlated with the target. When we have multiple variables having the same correlation, it extends in a direction that is equiangular (has the same angle) to the variables. It can compute the entire regularization path for approximately the same computational cost as a single least-squares fit.
- Lasso Least Angle Regression (Lasso LAR) [35]: this algorithm computes the Lasso path along the regularization using the Least Angle Regression algorithm. The Lasso parameters are solved using the Least Angle Regression algorithm, which yields piecewise linear solution paths as a function of the norm of its coefficients.
- Orthogonal Matching Pursuit (OMP) [36]: this algorithm starts the search by finding a column with maximum correlation with measurements at the first step, and then, at each iteration, it searches for the column with maximum correlation with current residual. The residuals after each step are orthogonal to all the selected columns. This algorithm is iteratively updated till a stopping criterion is met or the number of iterations passes a limit.
- Bayesian Ridge Regression [37]: this algorithm allows a natural mechanism to survive insufficient data or poorly distributed data by formulating linear regression using probability distributions rather than point estimates. It makes use of conjugate priors for the precision of the Gaussian and, because of that, is restricted to use gamma prior, which requires four hyperparameters chosen arbitrarily to be small values. It also requires initial values for parameters and alpha and lambda that are then updated from the data.
- Automatic Relevance Determination (ARD) [32]: this algorithm is very similar to the Bayesian Ridge Regression, but ARD makes the coefficients sparser. This is also known as sparse Bayesian learning and Relevance Vector Machine that ranks input variables in the order of their importance on predicting the output. It uses a parameterized, data-dependent prior distribution that effectively prunes away redundant or superfluous features.
- Passive Aggressive Regression (PAR) [38]: this algorithm is generally used for large-scale learning. It is one of the few online-learning algorithms. In online learning, the input data come in sequential order and the machine learning model is updated step by step, where the entire training dataset is used at once. This is suitable in situations where there is a large amount of data and it is computationally infeasible to train the entire dataset because of the sheer size of the data. If the prediction is correct, the model is kept and no changes are made (passive). If the prediction is incorrect, changes are made to the model (aggressive).
- Random Sample Consensus (RANSAC) [39]: this is an iterative method that is used to estimate parameters of a model from a set of data containing outliers. This algorithm assumes that all of the data consist of inliers and outliers. Inliers can be explained by a model with a particular set of parameter values, while outliers do not fit that model in any circumstance. This model can optimally estimate the parameters of the chosen model from the determined inliers.
- Huber Regression [40]: this is a regression method that is robust to outlier. It uses the Huber loss function rather than the least squares error. This function is identical to the least squares penalty for small residuals but, on large residuals, its penalty is lower and increases linearly rather than quadratically. It is, thus, more forgiving of outliers.

##### Nonlinear Model

- Multi-layer Perceptron (MLP) Regression [41]: this is an artificial neural network that has three or more layers of perceptrons. These layers are a single input layer, one or more hidden layers, and a single output layer of perceptrons. This has multiple layers of neurons with an activation function and a threshold value. Backpropagation is a technique where the multi-layer perceptron receives feedback on the error in its results and the MLP adjusts its weights accordingly to make more accurate prediction in the future.

#### 2.3.2. Nonparametric Algorithms

##### Linear Models

- Relevance Vector Regression (RVR) [2,14,16,22,42]: this is a Bayesian framework for learning sparse regression models. RVR has an identical functional form to SVR, but the Bayesian formulation of the RVR avoids the set of free parameters of the SVR. The sparsity of the RVR is induced by the hyperpriors on model parameters in a Bayesian framework, with the maximum a posteriori (MAP) principle. The behavior of the RVR is controlled by the type of kernel, which has to be selected, while all other parameters are automatically estimated by the learning procedure itself.
- Theil–Sen Regression [43]: this algorithm is a nonparametric method that determines the slope of the regression line via the median of the slopes of all lines that can be drawn through the data points. Alternative to least squares for simple linear regression, it uses a generalization of the median in multiple dimensions and is, thus, robust to multivariate outliers.

##### Nonlinear Models

- Support Vector Regression (SVR) [14,15,16,23,44]: this is characterized by the use of kernels, sparsity, control of the margin of tolerance (epsilon, ε), and the number of support vectors. SVR supports both linear and nonlinear regression. A kernel helps us find a hyperplane in the higher dimensional space without increasing the computation cost. This algorithm constructs a hyperplane or a set of hyperplanes in a high or even infinite dimensional space. There are two lines that are drawn around the hyperplane at a distance of ε, which is used to create a margin between the data points. It identifies a symmetrical ε-insensitive region (ε-tube). We can choose any kernel, such as sigmoid kernel, polynomial kernel, and radial basis function kernel. A linear kernel was chosen for SVR.
- Gaussian Processes Regression (GPR) [8,16,45]: this is a nonparametric kernel-based probabilistic approach. GPR model can make predictions incorporating prior knowledge (kernels) and provide uncertainty measures over predictions. The Gaussian processes conduct regression by defining a distribution over an infinite number of functions.
- Kernel Ridge Regression (KRR) [32]: this algorithm combines Ridge Regression with the kernel trick. It uses squared error loss, whereas Support Vector Regression uses ε-insensitive loss, both combined with L2 regularization. A polynomial kernel was chosen for KRR.
- K-Nearest Neighbors (kNN) Regression [15,46]: this algorithm uses feature similarity to predict the values of any new data points, which means that the new point is assigned a value based on how closely it resembles the points in the training set. This method uses Euclidean distance to find the nearest neighbors to an object. The closest “k” data points are selected based on the distance. The average value of these data points is the final prediction for the new point.

##### Ensemble Models

- Decision Tree (DT) Regression [41]: this is a decision-making algorithm that uses a flowchart-like tree structure. This algorithm observes features of an object that train a model in the structure of a tree to predict data in the future to produce meaningful continuous output. Starting from a root node, it builds a decision tree with decision nodes and leaf nodes, which employs a top-down, greedy search through the space of possible branches with no backtracking. A decision tree is built top-down from a root node and involves partitioning the data into subsets that contain instances with similar values.
- Random Forest (RF) Regression [15,47]: this is a supervised learning algorithm that uses ensemble learning method for regression. It operates by constructing multiple decision trees during training time and determining the final output rather than relying on individual decision trees. Each tree is constructed by bootstrapping that performs row sampling and features a sample from the dataset. The final output is the mean of all the outputs (aggregation).
- Extra Trees (ET) Regression [48]: this is an ensemble machine learning algorithm that combines the predictions from many decision trees. It is similar to other methods, such as decision trees and random forests, but it uses extra information about the data to improve predictive accuracy. This method aggregates the results of multiple decorrelated decision trees collected in a forest to output. Random forests use bootstrapping that subsamples the input data with replacement, whereas extra trees use the entire original dataset. In terms of the selection of cut-points to split nodes, random forests choose the optimum split, while extra trees choose it randomly.
- Adaptive Boosting (AdaBoost) Regression [49,50]: this algorithm involves using very short (one-level) decision trees as weak learners that are added sequentially to the ensemble. This is a boosting ensemble algorithm where models are added sequentially and later models in the sequence correct the predictions made by earlier models in the sequence.
- Multi-layer Perceptron (MLP) Regression [41]: this is an artificial neural network that has three or more layers of perceptrons. These layers are a single input layer, one or more hidden layers, and a single output layer of perceptrons. This has multiple layers of neurons with an activation function and a threshold value. Backpropagation is a technique where the multi-layer perceptron receives feedback on the error in its results and the MLP adjusts its weights accordingly to make more accurate prediction in the future.
- Gradient Boosting Machine (GBM) [51]: this is an ensemble algorithm that fits boosted decision trees by minimizing an error gradient. Models are fit using any arbitrary differentiable loss function and gradient descent optimization algorithm. The general concept of gradient boosting and adaptive boosting is essentially the same: they are both ensemble models boosting (stacking) trees on top of each other based on the model mistakes. The main difference is that, in gradient boosting, each new weak learner is stacked directly on the model’s current errors rather than on a weighted version of the initial training set.
- Extreme Gradient Boosting (XGBoost) [52]: this is an optimized distributed gradient boosting algorithm designed to be highly efficient, flexible, and portable. Both XGBoost and gradient bosting algorithm are ensemble tree methods that apply the principle of boosting weak learners using the gradient descent architecture. However, XGBoost improves upon the base gradient boosting framework through systems optimization and algorithmic enhancements.
- Light Gradient Boosting Machine (LightGBM) [53]: this extends the gradient boosting algorithm by adding a type of automatic feature selection and focusing on boosting examples with large gradients. It is based on decision trees to increase the efficiency of the model and reduces memory usage using gradient-based one side sampling (GOSS) and exclusive feature bundling (EFB), which fulfills the limitations of a histogram-based algorithm.
- Category Boosting (CatBoost) Regression [54]: this algorithm is another member of the gradient boosting technique on decision trees. CatBoost provides an inventive method for processing categorical features, based on target encoding. This method, named ordered target statistics, tries to solve a common issue that arises when using such a target encoding, which is target leakage. It uses oblivious decision trees, where the same splitting criterion is used across an entire level of the tree. Such trees are balanced, less prone to overfitting, and allow speeding up prediction significantly at testing time.

### 2.4. Brain Age Prediction Framework

We applied 27 machine learning algorithms separately to each sample (HCP, Cam-CAN, and IXI) using identical procedures. Each of the samples was divided into a training set (80%) and a test set (20%) by a conditionally random method, such that the distributions of age and sex in the two sets were statistically identical. Details about sample and demographic information for the three samples are provided in Supplementary Table S3. Prior to building a model, each morphological measure was standardized so that the data have a mean of zero and a standard deviation of one. For each algorithm, we tuned hyperparameters using 10-fold cross-validation to learn the model parameters and evaluate the model. Each algorithm was trained using grid search to find the best parameters that give the highest accuracy. The performance of each algorithm was quantified by the Pearson’s correlation coefficient (r) and mean absolute error (MAE) between predicted brain age and chronological age [6]. We also reported weighted MAE for comparison between studies with different sample age ranges. We divided the MAE value by the age range of the hold-out test set to calculate the weighted MAE value [13]. Finally, computational efficiency for each algorithm was assessed by recording the total computational time to train the model via 10-fold cross-validation on the training data. All models were implemented in Python and trained on a machine with AMD Ryzen 9 5900X CPU and 32 GB RAM.

### 2.5. Age-Bias Correction

BrainPAD was computed for each algorithm by subtracting the chronological age of each individual from their brain age predicted by that algorithm. BrainPAD is often overestimated in younger individuals and underestimated in older individuals due to general statistical features of the regression analysis [55]. To account for age bias, we used an approach introduced by de Lange and colleagues [6]. A correction procedure was applied by using Y=αΩ+β, where Y is the modeled predicted age as a function of chronological age (Ω), and α and β denote the slope and intercept, respectively. The α and β coefficients form a linear fit and were used to correct predicted brain age with “corrected predicted brain age” = “predicted brain age” + [Ω−(αΩ+β)]. A bias-free brainPAD was then calculated as “corrected brainPAD” = “corrected predicted brain age” − “chronological age”.

### 2.6. Comparative Evaluation of the Algorithms

We performed comparative evaluation of the algorithms within each sample, separately (i.e., HCP, Cam-CAN, and IXI) based on the within-sample similarity in predicted brain age using the Pearson’s correlation analyses and hierarchical clustering with Ward’s minimum variance methods for Euclidian distances [2]. The statistical comparison of algorithms was performed by an analysis of variance (ANOVA) test followed by post hoc analyses using Tukey’s honestly significant difference at a significance level of 5%.

### 2.7. Feature Importance

To identify the contribution of individual morphological features to brain age prediction, we chose three different model types of models with high accuracy (one for linear model, one for nonlinear model, and one for ensemble model). For each of the three best performing algorithms, we employed kernel Shapley additive explanation (SHAP) [56] to examine regional morphological features that contribute to model prediction error (or brainPAD). For each sample, we estimated the SHAP values to identify important features in the three selected models separately. The sum of SHAP values across all features is equal to the difference between the predicted output and the expected model output from the entire training data. Here, we defined the baseline set using 10 nearest neighbors in the training sample to compute age-specific feature importance values for each test subject [57]. This resulted in a “model error explanation” matrix with size of subject × feature, where each column represents the importance of a given regional feature to an individual’s brainPAD, relative to the age-matched training samples, and each row reflects an individual’s feature importance. The sum of all SHAP values across features corresponds to the individual’s model prediction error or brainPAD.

## 3. Results

### 3.1. Algorithm Performance for Brain Age Prediction

The performance for each of the 27 algorithms in the HCP, Cam-CAN, and IXI samples are shown in Table 1, Table 2 and Table 3 for the training sets (model performance) and the hold-out test sets (prediction performance). The prediction accuracy varied by regression algorithms. Correlations between chronological age and predicted brain age across 27 algorithms for each of the three samples are provided in detail in Supplementary Figures S2–S4.

In the HCP, the MAE values ranged between 2.75 and 3.12 (weighted MAE = 0.18–0.21) and the r values ranged between 0.11 and 0.43. The highest and lowest prediction accuracies were achieved by Lasso (MAE = 2.75; r = 0.43) and decision tree (MAE = 3.12; r = 0.11), respectively. In the Cam-CAN, the MAE values ranged between 7.08 and 10.50 (weighted MAE = 0.10–0.15) and the r values ranged between 0.64 and 0.86. The highest and lowest prediction accuracies were achieved by Lasso LAR (MAE = 7.08; r = 0.86) and decision tree (MAE = 10.50; r = 0.64), respectively. In the IXI, the MAE values ranged between 8.04 and 9.86 (weighted MAE = 0.12–0.15) and the r values ranged between 0.63 and 0.80. The highest and lowest prediction accuracies were achieved by ARD (MAE = 8.04; r = 0.80) and decision tree (MAE = 9.86; r = 0.63), respectively. Overall, the regularized linear models (e.g., Lasso, Lasso LAR, and ARD), followed by the ensemble models (e.g., GBM, CatBoost, and LightGBM), achieved a good performance in the hold-out test sets across three samples. In spite of nominal ranking of the algorithms, the top 10 algorithms performed comparably well (Table 1, Table 2 and Table 3). Specifically, based on algorithm performance, we identified three different model types of models, namely Lasso for regularized linear model, GPR for nonlinear model, and GBM for ensemble model, which we evaluated further for quantifying feature importance in the subsequent section.

### 3.2. Comparative Performance of the Algorithms for Brain Age Prediction

**Figure 1:**
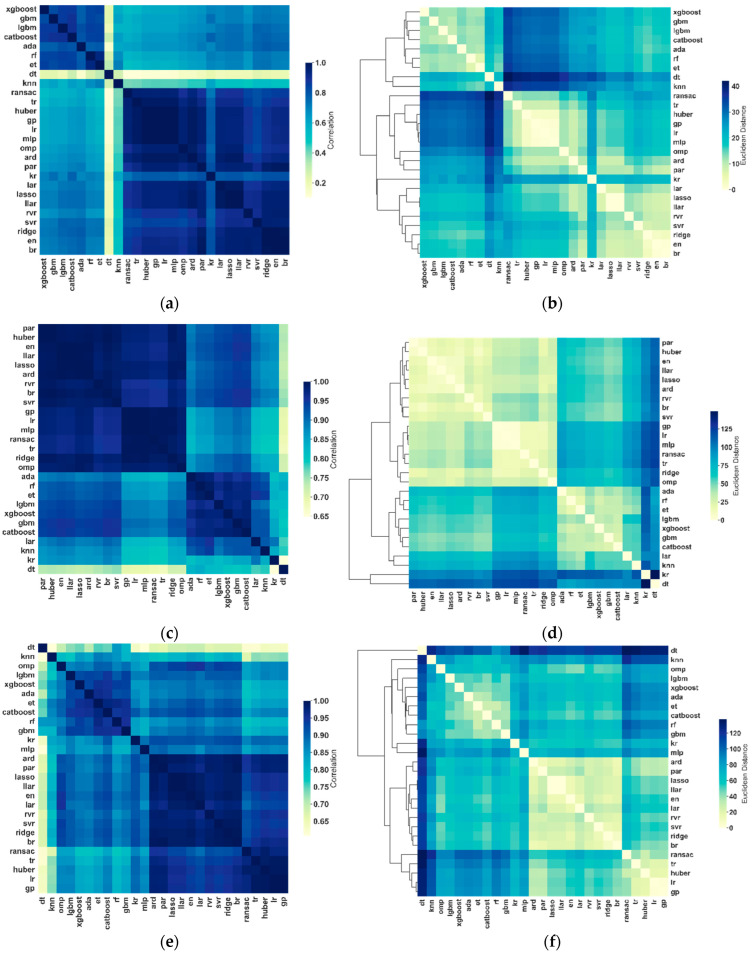
Similarity in predicted brain age in the hold-out test sets for the HCP, Cam-CAN, and IXI samples across 27 algorithms. For the HCP sample, (**a**) similarity matrix representing between-algorithm correlations of individual predicted brain age and (**b**) distance matrix and dendrogram resulting from hierarchical clustering of the individual brain age results of the 27 algorithms. For the Cam-CAN sample, (**c**) similarity matrix representing between-algorithm correlations of individual predicted brain age and (**d**) distance matrix and dendrogram resulting from hierarchical clustering of the individual brain age results of the 27 algorithms. For the IXI sample, (**e**) similarity matrix representing between-algorithm correlations of individual predicted brain age and (**f**) distance matrix and dendrogram resulting from hierarchical clustering of the individual brain age results of the 27 algorithms. lasso = Least Absolute Shrinkage and Selection Operator; llar = Lasso Least Angle Regression; svr = Support Vector Regression; lar = Least Angle Regression; en = Elastic Net Regression; br = Bayesian Ridge Regression; ridge = Ridge Regression; ard = Automatic Relevance Determination; rf = Random Forest Regression; par = Passive Aggressive Regression; cat = Category Boosting Regression; rvr = Relevance Vector Regression; lgbm = Light Gradient Boosting Machine; gbm = Gradient Boosting Machine; knn = K-Nearest Neighbors; ada = Adaptive Boosting Regression; et = Extra Trees Regression; xgb = Extreme Gradient Boosting; kr = Kernel Ridge Regression; gp = Gaussian Processes Regression; mlp = Multi-layer Perceptron Regression; omp = Orthogonal Matching Pursuit; lr = Linear Regression; huber = Huber Regression; tr = Theil–Sen Regression; ransac = Random Sample Consensus; dt = Decision Tree Regression.

In the HCP, pairwise correlations in predicted brain ages between algorithms ranged from 0.1 to 0.97 (Table 1; Figure 1a). Hierarchical clustering of the individual predicted brain ages identified three clusters (Figure 1b): ensemble models and kNN formed one cluster, GPR, MLP, and the seven linear models formed another cluster (RANSAC, Theil–Sen, Huber, Linear Regression, OMP, ARD, and PAR), and kernel ridge regression and the eight linear models (LAR, Lasso, Lasso LAR, RVR, SVR, Ridge, Elastic Net, and Bayesian Ridge) formed a third cluster. In the Cam-CAN, pairwise correlations in predicted brain ages between algorithms ranged from 0.64 to 0.99 (Table 2; Figure 1c). Hierarchical clustering of the individual predicted brain ages identified four clusters (Figure 1d): one cluster included the nine linear models (PAR, Huber, Elastic Net, Lasso LAR, Lasso, ARD, RVR, Bayesian Ridge, and SVR). GPR, MLP, and the five linear models (LR, RANSAC, Theil–Sen, Ridge, and OMP) formed another cluster. KNN, LAR, and ensemble models formed a third cluster. Kernel ridge and decision tree regressions together formed a fourth cluster, where decision tree showed the lowest similarity with all the other algorithms. In the IXI, pairwise correlations in predicted brain ages between algorithms ranged from 0.6 to 0.99 (Table 3; Figure 1e). In Figure 1f, hierarchical clustering analyses showed that ensemble models, kNN, OMP, kernel ridge, and MLP formed one cluster. A second cluster included the 10 linear models (ARD, PAR, Lasso, Lasso LAR, Elastic Net, LAR, RVR, SVR, Ridge, and Bayesian Ridge). GPR and the four linear models (RANSAC, Theil–Sen, Huber, and LR) formed a third cluster. Similarly, decision tree regression showed the lowest similarity with all the other algorithms, as shown in the HCP and Cam-CAN samples.

### 3.3. Computational Speed of the Algorithms

The total computation time to train the model using 10-fold cross-validation for each algorithm is shown in Table 4. Among algorithms, Ridge (0.06 ± 0.01 s) and OMP (0.07 ± 0.01 s) were the fastest algorithms, whereas Theil–Sen (58.31 ± 0.59 s) and CatBoost (45.97 ± 1.28 s) were the slowest algorithms. As expected, the linear algorithms (0.06–4.87 s), with the exception of Theil–Sen, took less than the ensemble models (0.07–47.67 s) for model training. Most of the linear algorithms, such as Lasso, OMP, LAR, and PAR, took less than 1 s. Among ensemble algorithms, LightGBM took less than 1 s, while CatBoost took the longest training time (45.97 ± 1.28 s).

### 3.4. Comparison of the BrainPAD of the Algorithms

**Figure 2:**
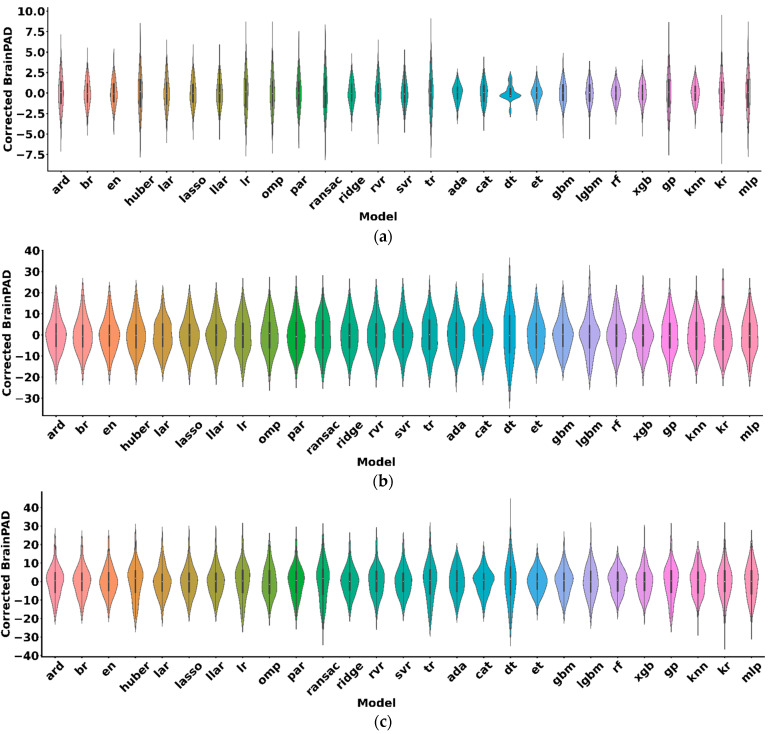
Corrected brainPAD (corrected predicted brain age–chronological age) in the HCP, Cam-CAN, and IXI samples. Violin plots showing the distributions of individual corrected brainPAD values in the hold-out test sets for the (**a**) HCP, (**b**) Cam-CAN, and (**c**) IXI samples. Box plot within each violin plot shows the first quartile (Q1) and third quartile (Q3) of the corrected brainPAD values. White circle within each boxplot indicates the median corrected brainPAD value. lasso = Least Absolute Shrinkage and Selection Operator; llar = Lasso Least Angle Regression; svr = Support Vector Regression; lar = Least Angle Regression; en = Elastic Net Regression; br = Bayesian Ridge Regression; ridge = Ridge Regression; ard = Automatic Relevance Determination; rf = Random Forest Regression; par = Passive Aggressive Regression; cat = Category Boosting Regression; rvr = Relevance Vector Regression; lgbm = Light Gradient Boosting Machine; gbm = Gradient Boosting Machine; knn = K-Nearest Neighbors; ada = Adaptive Boosting Regression; et = Extra Trees Regression; xgb = Extreme Gradient Boosting; kr = Kernel Ridge Regression; gp = Gaussian Processes Regression; mlp = Multi-layer Perceptron Regression; omp = Orthogonal Matching Pursuit; lr = Linear Regression; huber = Huber Regression; tr = Theil–Sen Regression; ransac = Random Sample Consensus; dt = Decision Tree Regression.

Figure 2 shows the distributions of individual corrected brainPAD values in the hold-out test sets for the HCP, Cam-CAN, and IXI samples. Statistical analyses revealed that none of the algorithms show significant differences in corrected brainPAD between algorithms (F = 8 × 10^−20^, *p* > 0.05 for the HCP; F = 7 × 10^−29^, *p* > 0.05 for the Cam-CAN; F = 1 × 10^−28^, *p* > 0.05 for the IXI). Nevertheless, we observed a substantial variation in corrected brainPAD: the range of corrected brainPAD for the HCP, Cam-CAN, and IXI samples was −7.17–8.08, −25.40–40.22, and −29.69–36.10, respectively. In particular, in the HCP, decision tree had the narrowest brainPAD range of −2.32–2.01, whereas RANSAC had the broadest brainPAD range of −6.41–6.61. In the Cam-CAN, GBM had the narrowest brainPAD range of −15.73–19.75, whereas decision tree had the broadest brainPAD range of −25.40–27.16. In the IXI, extra trees had the narrowest brainPAD range of −16.02–15.72, whereas decision tree had the broadest brainPAD range of −26.06–36.10.

### 3.5. Regional Contributions to Brain Age Prediction

**Figure 3:**
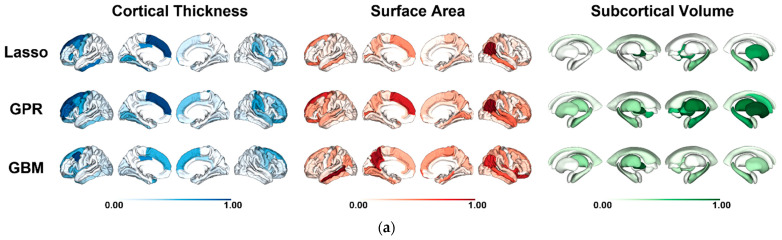

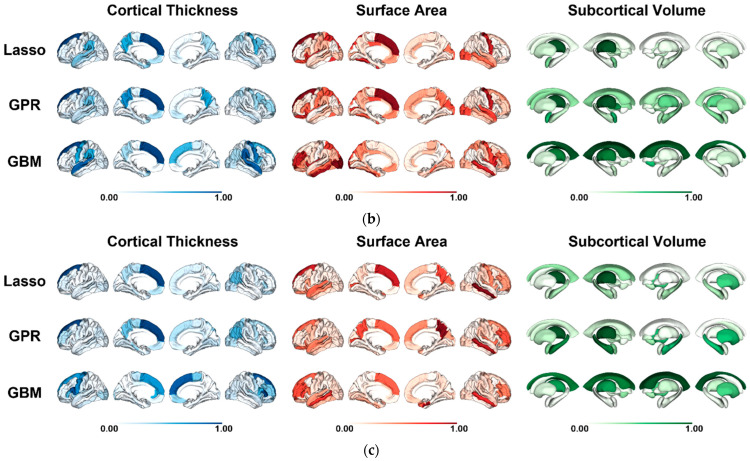
SHAP feature importance quantified as the mean absolute SHAP value for the (**a**) HCP, (**b**) Cam-CAN, and (**c**) IXI samples. Mean absolute feature importance (SHAP value) averaged across all subjects for regional cortical thickness, surface area, and subcortical volume for Least Absolute Shrinkage and Selection Operator (Lasso) Regression, Gaussian Process Regression (GPR), and Gradient Boosting Machine (GBM). Darker colors indicate higher feature importance in the explanation of model prediction error or brainPAD. The relative feature importance values shown are rescaled such that the feature with the maximum average absolute SHAP value in each model is assigned a value of 1. The top 20 regional features for all models are shown in Supplementary Tables S4–S6.

We estimated SHAP values to examine to what extent regional features contribute to brain age prediction error or brainPAD. Figure 3 shows the regional feature importance to brainPAD for each model, based on mean absolute SHAP values averaged across subjects. In the HCP, features with the highest average contribution to brainPAD for all models included total intracranial volume, cortical thickness of regions in the left superior frontal gyrus and the left caudal middle fontal gyrus, surface area of regions in the right inferior parietal lobule, as well as subcortical regions in the left pallidum and right putamen. In the Cam-CAN, features with the highest average contribution to brainPAD for all models included total intracranial volume, cortical thickness of regions in the left superior frontal gyrus, the left precuneus, and the left supramarginal gyrus, surface area of regions in the left superior fontal gyrus and the right precentral gyrus, as well as subcortical regions in the left thalamus and the left amygdala. In the IXI, features with the highest average contribution to brainPAD for all models included total cranial volume, cortical thickness of regions in the left superior frontal gyrus and the right pars triangularis, and surface area of regions in the right middle temporal gyrus, as well as subcortical regions in left thalamus and right putamen. The top 20 regional features for all models are shown in Supplementary Tables S4–S6.

For each sample, we compared three different types of model, namely Lasso, GPR, and GBM. Across all samples, a high correspondence in average feature importance was observed between Lasso and GPR (r = 0.89–0.95). We also found a moderate correlation between Lasso and GBM (r = 0.3–0.55) and a low similarity between GPR and GBM (r = 0.17–0.38). Details about pairwise correlations between models for each sample are provided in Supplementary Figure S5. Furthermore, supplemental analyses for the three algorithms were also conducted to examine the effects of feature selection on regression performance (see Supplementary Materials for more details).

## 4. Discussion

In this study, we applied 27 different machine learning algorithms based on brain morphological features to predict brain age. We conducted a comprehensive evaluation of machine learning algorithms using three different independent datasets. We demonstrated that different machine learning algorithms applied to the same brain morphological data led to a substantial variation in predicted brain age. This finding was replicated across three datasets and 27 regression algorithms.

Our previous study showed that brain age prediction with the morphological features was substantially influenced by the choice of algorithm [2]. In this study, we expanded our prior work by evaluating 27 machine learning algorithms and showing computational efficiency for each algorithm. We also replicated our prior results not just in the young adult HCP participants, but also in the Cam-CAN and the IXI datasets, which focused on elderly participants. We found that algorithm choice yielded variations in brain age estimates despite being applied to the same morphological data. In the HCP, the models achieved an MAE of between 2.75 and 3.12 and a correlation coefficient of between 0.11 and 0.42. In the Cam-CAN dataset, the models achieved an MAE of between 7.08 and 10.50 and a correlation coefficient of between 0.64 and 0.85. In the IXI dataset, the models achieved an MAE of between 8.04 and 9.86 and a correlation coefficient of between 0.63 and 0.79. Across three datasets, we found a similar trend that the performance of the regularized linear regression models (weighted MAE = 0.10–0.20) were as good as the nonlinear and ensemble regression models (weighted MAE = 0.11–0.20). Our results showed that Lasso LAR, Lasso, and ARD performed best but there were minimal differences in accuracy when comparing with other ensemble models. Our results showed that the ensemble models are not always better than the regularized regression models. The regularized algorithms tend to make the coefficients sparser by shrinking irrelevant feature weights to zero, so the brain age prediction was performed based on relatively few brain morphological features. Moreover, model complexity can be controlled by including the regularization (or penalty) term in the models (e.g., L1-norm for Lasso, L2-norm for Ridge, and both L1-norm and L2-norm for Elastic Net). This helps the models less vulnerable to the collinearity among the predictor variables [2]. Meanwhile, ensemble methods can be useful in reducing variance and making more robust models. The aggregated results of multiple models are always less noisy than the individual models, which leads to model stability and robustness. However, using ensemble methods reduces model interpretability due to increased complexity. The ensemble models perform better when the predictors are independent. As a consequence, the performance of the regularized models was similar to that of the ensemble models. Decision tree algorithms achieved the lowest accuracies across all samples. Evaluation of the 27 regression models in three sizable samples of healthy individuals from the HCP, Cam-CAN, and IXI yielded reproducible results with regards to the similarity among the linear regression models (e.g., Lasso, Lasso LAR, RVR, and SVR), as well as among the ensemble models (e.g., AdaBoost, CatBoost, GBM, LightGBM, RF, and XGBoost) that consistently clustered together. Individual brain age predicted by decision tree was least correlated with all the other regression algorithms. We also evaluated the machine learning models for their sensitivity to different sample characteristics. We found differences in accuracy due to different age range in the test sample. These results indicate that model generalizability to unseen samples is likely sensitive to the age composition in the sample [6].

There are relatively few studies comparing between brain age prediction models with brain morphological features. Two recent studies undertook comparative evaluations of several machine learning algorithms on the basis of brain morphological data. Valizadeh et al. examined the performance of six algorithms, namely Multiple Linear Regression, Ridge Regression, Neural Network, K-Nearest Neighbors, Support Vector Regression, and Random Forests, in 3144 healthy participants from multiple cohorts, aged 7–96 years [15]. They reported that Multiple Linear Regression approach with a smaller set of morphological measures, consisting of only 11 larger brain regions, resulted in a higher prediction accuracy (*R*^2^ = 0.73). They also showed that Neural Network approach performed best based on a combination of different morphological features (*R*^2^ = 0.83). Baecker et al. tested the performance of three algorithms tested here, namely Support Vector Regression, Relevance Vector Regression, and Gaussian Process Regression, in 10,824 participants in the UK Biobank, aged 47–73 years [16]. They reported minimal differences in accuracy with the MAE values, ranging from 3.7 to 4.7 years, in the three algorithms tested. Our results showed that prediction accuracies with regularized linear regression models across three datasets (weighted MAE = 0.10–0.20) only marginally differed from those with ensemble regression models (weighted MAE = 0.11–0.20). These results are in line with a previous study, which showed that a simple multiple linear regression model with fewer morphological features achieved a good performance in prediction accuracy [15]. Moreover, in 768 typically developing children and adolescents (aged 3–21 years), Elastic Net Regression, Gaussian Process Regression, and XGBoost applied to the same cortical features had similar performance (MAE = 1.75–1.92; *R*^2^ = 0.78–0.81) in brain age prediction [57]. Thus, it may not be necessary to use more complicated, computationally expensive models (e.g., tree-ensemble model types) to achieve accurate brain age prediction when using the morphological features as input data. It is worth noting that the regularized linear models offer good performance in brain age prediction, with low computational costs.

We applied the kernel SHAP approach to three different model types (Lasso, GPR, and GBM) for the purpose of estimating individual-level explanations for model-predicted error (brainPAD). This helps understand to what extent different regional features contribute to brainPAD across all three different model types [57]. We chose three representative regression models with high accuracy, since these have been widely used approaches for brain age prediction [8,14,15,22,23,57] and provided a high prediction accuracy. In the regularized linear models, the regularization parameters (e.g., L1-norm for Lasso Regression, L2-norm for Ridge Regression) make the models have fewer features, so that brain age prediction models with linear algorithms are more simple and interpretable. On the other hand, complex regression models with nonlinear combinations of features are less interpretable. In three datasets, we showed the relative contribution of each brain region to brain age prediction for each of the three selected models. Our SHAP analyses revealed a similar correspondence between Lasso and GPR (r = 0.89–0.95) but a low similarity between GPR and GBM (r = 0.17–0.38). Multiple morphological features were identified as predictive regions for brain age prediction (Figure 3; Supplementary Tables S4–S6). Overall, the most important features that explain brainPAD were total intracranial volume, cortical thickness of frontal (superior frontal gyrus, caudal middle frontal gyrus, and pars triangularis) and parietal regions (precuneus and supramarginal gyrus), and surface area of regions in the superior frontal gyrus, the lateral orbitofrontal gyrus, and the middle temporal gyrus. The features least contributing to brainPAD were cortical thickness of region in the caudal anterior cingulate cortex and surface area of region in the parahippocampal gyrus. However, we note that our results were partly inconsistent with that of Ball and colleagues, who found that the contributions of cortical features (cortical thickness and surface area) that explain model predictions were consistent across model types [57]. One possible reason for this discrepancy might be due to the choice of model types (Elastic Net Regression, GPR, and XGBoost). Another possible reason might be related to differences between sample composition. They found a high correspondence in average feature importance across different model types in typically developing children (age = 3–21 years) [57]. Other reasons might be parcellation choice and the exclusion of subcortical volume as input feature.

We acknowledge several limitations that could be addressed in future studies. The focus of this study was on the evaluation of the different machine learning algorithms in predicting brain age on the basis of brain morphological features, not on the examination of functional significance of brainPAD on behavioral and clinical scores [3,4,5,8,12,22,23,58]. Nevertheless, individualized prediction of brain age presented in this work can be easily used to calculate brainPAD (predicted brain age–chronological age), and then applied to test for its association with behavioral and clinical scores in clinical populations [3,4,5,8,12,22,23,58]. In this study, we have focused on brain age prediction using the 68 cortical regions of interest (ROIs) from the Desikan-Killiany parcellation, as well as the 16 subcortical volumes, which is a widely used approach in larger neuroimaging studies [30]. Future research should replicate the current findings in independent datasets, across different atlases and at different spatial resolutions (e.g., the Schaefer parcellation [59]). Here, we have shown that different machine learning models applied to the same anatomical features yielded variations in predicted brain age across three different samples. However, several studies have started to explore the value of multimodal brain age prediction performance in healthy participants and in disease populations, showing improved prediction of clinical markers with multimodal imaging [11,23]. The benefits of multimodal imaging could be further examined in future work, focusing on the identification of disease and aging markers that can benefit from multimodal imaging, and comparing the utility of each modality in predicting these markers. Finally, we showed the performance of different machine learning models that provide a good coverage of many models that are presently available. It is also important to note that deep learning models have surpassed classical machine learning approaches in brain age prediction. Previous studies have proved that brain age estimation using deep learning algorithms, such as simple fully convolutional network (SFCN) [60], deep brain network (DeepBrainNet) [58], and attention-driven multi-channel fusion neural network (FiA-Net) [61], outperform traditional machine learning methods when processing large and diverse MRI datasets. Future work could assess advanced deep learning models but at the cost of interpretability and model complexity.

## 5. Conclusions

Through applying 27 machine learning algorithms to the same morphological features in the HCP, Cam-CAN, and IXI datasets, we showed that algorithm choice introduces a substantial variation in predicted brain age. By evaluating various regression models for brain age prediction across three independent datasets, we showed that the regularized linear models might be just as effective as the nonlinear and ensemble models for predicting brain age based on brain morphological features, while significantly reducing computational costs.

## Figures and Tables

**Table 1:** Algorithm performance based on the structural features from the HCP individuals entered in the model for model performance in the training data (*n* = 890) and prediction performance in the hold-out test data (*n* = 223).

Algorithm	Model Performance			Prediction Performance		
	r	MAE	Weighted MAE	r	MAE	Weighted MAE
Lasso	0.4921	2.6444	0.1763	0.4258	2.7565	0.1838
Lasso LAR	0.4921	2.6444	0.1763	0.4258	2.7565	0.1838
SVR	0.4515	2.6981	0.1799	0.4268	2.7756	0.1850
LAR	0.4723	2.6933	0.1796	0.4124	2.7896	0.1860
Elastic Net	0.4714	2.6737	0.1782	0.4199	2.7919	0.1861
Bayesian Ridge	0.4712	2.6745	0.1783	0.4182	2.7927	0.1862
Ridge	0.4698	2.6797	0.1786	0.4255	2.7941	0.1863
ARD	0.4973	2.6373	0.1758	0.3991	2.8251	0.1883
Random Forest	0.4245	2.7785	0.1852	0.4131	2.8304	0.1887
PAR	0.4563	2.7231	0.1815	0.4010	2.8322	0.1888
CatBoost	0.4282	2.7631	0.1842	0.4069	2.8328	0.1889
RVR	0.4498	2.7148	0.1810	0.4021	2.8371	0.1891
LightGBM	0.4273	2.7457	0.1830	0.4016	2.8418	0.1895
GBM	0.4458	2.7149	0.1810	0.4000	2.8437	0.1896
kNN	0.3768	2.8367	0.1891	0.3801	2.8591	0.1906
AdaBoost	0.3982	2.8003	0.1867	0.4188	2.8595	0.1906
Extra Trees	0.4224	2.7738	0.1849	0.4197	2.8674	0.1912
XGBoost	0.4201	2.7726	0.1848	0.3859	2.8771	0.1918
Kernel Ridge	0.4417	2.7495	0.1833	0.3878	2.8775	0.1918
GPR	0.4735	2.7199	0.1813	0.3689	2.9420	0.1961
MLP	0.4744	2.7216	0.1814	0.3675	2.9450	0.1963
OMP	0.4790	2.6927	0.1795	0.3590	2.9457	0.1964
LR	0.4736	2.7244	0.1816	0.3679	2.9474	0.1965
Huber	0.4705	2.7366	0.1824	0.3674	2.9484	0.1966
Theil–Sen	0.4663	2.7544	0.1836	0.3398	2.9724	0.1982
RANSAC	0.4553	2.8094	0.1873	0.3627	3.0015	0.2001
Decision Tree	0.1694	3.0653	0.2044	0.1122	3.1206	0.2080

Lasso = Least Absolute Shrinkage and Selection Operator; Lasso LAR = Lasso Least Angle Regression; SVR = Support Vector Regression; LAR = Least Angle Regression; Elastic Net = Elastic Net Regression; Bayesian Ridge = Bayesian Ridge Regression; Ridge = Ridge Regression; ARD = Automatic Relevance Determination; Random Forest = Random Forest Regression; PAR = Passive Aggressive Regression; CatBoost = Category Boosting Regression; RVR = Relevance Vector Regression; LightGBM = Light Gradient Boosting Machine; GBM = Gradient Boosting Machine; kNN = K-Nearest Neighbors; AdaBoost = Adaptive Boosting Regression; Extra Trees = Extra Trees Regression; XGBoost = Extreme Gradient Boosting; Kernel Ridge = Kernel Ridge Regression; GPR = Gaussian Processes Regression; MLP = Multi-layer Perceptron Regression; OMP = Orthogonal Matching Pursuit; LR = Linear Regression; Huber = Huber Regression; Theil–Sen = Theil–Sen Regression; RANSAC = Random Sample Consensus; Decision Tree = Decision Tree Regression.

**Table 2:** Algorithm performance based on the structural features from the Cam-CAN individuals entered in the model for model performance in the training data (*n* = 500) and prediction performance in the hold-out test data (*n* = 101).

Algorithm	Model Performance			Prediction Performance		
	r	MAE	Weighted MAE	r	MAE	Weighted MAE
Lasso LAR	0.8952	6.6767	0.0954	0.8589	7.0830	0.1012
ARD	0.8992	6.5372	0.0934	0.8585	7.1040	0.1015
Lasso	0.8943	6.6898	0.0956	0.8567	7.1757	0.1025
Elastic Net	0.8960	6.6632	0.0952	0.8548	7.1816	0.1026
Huber	0.8938	6.7060	0.0958	0.8455	7.4663	0.1067
Bayesian Ridge	0.8927	6.7691	0.0967	0.8445	7.4698	0.1067
RVR	0.8824	6.9355	0.0991	0.8378	7.5311	0.1076
PAR	0.8877	6.9834	0.0998	0.8395	7.5762	0.1082
Ridge	0.8906	6.8230	0.0975	0.8432	7.5865	0.1084
OMP	0.8827	7.0357	0.1005	0.8437	7.6179	0.1088
GPR	0.8839	7.0175	0.1003	0.8377	7.7190	0.1103
LR	0.8826	7.0582	0.1008	0.8366	7.7432	0.1106
MLP	0.8831	7.0570	0.1008	0.8364	7.7450	0.1106
SVR	0.8887	6.8523	0.0979	0.8309	7.7551	0.1108
RANSAC	0.8789	7.2202	0.1031	0.8282	7.8652	0.1124
Theil–Sen	0.8791	7.1771	0.1025	0.8366	7.8698	0.1124
GBM	0.8681	7.3435	0.1049	0.8368	7.9222	0.1132
CatBoost	0.8667	7.3767	0.1054	0.8230	8.1285	0.1161
XGBoost	0.8552	7.5686	0.1081	0.8167	8.3920	0.1199
LightGBM	0.8646	7.1822	0.1026	0.8040	8.4686	0.1210
Kernel Ridge	0.876	7.2091	0.1030	0.7022	8.6938	0.1242
Extra Trees	0.8565	7.7800	0.1111	0.8050	8.8377	0.1263
Random Forest	0.8410	8.0043	0.1143	0.7955	8.9883	0.1284
AdaBoost	0.8405	8.0458	0.1149	0.7725	9.4055	0.1344
LAR	0.8378	8.3740	0.1196	0.7577	9.5307	0.1362
kNN	0.8234	8.7403	0.1249	0.7709	9.6734	0.1382
Decision Tree	0.7259	9.7473	0.1392	0.6430	10.5017	0.1500

Lasso = Least Absolute Shrinkage and Selection Operator; Lasso LAR = Lasso Least Angle Regression; SVR = Support Vector Regression; LAR = Least Angle Regression; Elastic Net = Elastic Net Regression; Bayesian Ridge = Bayesian Ridge Regression; Ridge = Ridge Regression; ARD = Automatic Relevance Determination; Random Forest = Random Forest Regression; PAR = Passive Aggressive Regression; CatBoost = Category Boosting Regression; RVR = Relevance Vector Regression; LightGBM = Light Gradient Boosting Machine; GBM = Gradient Boosting Machine; kNN = K-Nearest Neighbors; AdaBoost = Adaptive Boosting Regression; Extra Trees = Extra Trees Regression; XGBoost = Extreme Gradient Boosting; Kernel Ridge = Kernel Ridge Regression; GPR = Gaussian Processes Regression; MLP = Multi-layer Perceptron Regression; OMP = Orthogonal Matching Pursuit; LR = Linear Regression; Huber = Huber Regression; Theil–Sen = Theil–Sen Regression; RANSAC = Random Sample Consensus; Decision Tree = Decision Tree Regression.

**Table 3:** Algorithm performance based on the structural features from the IXI individuals entered in the model for model performance in the training data (*n* = 453) and prediction performance in the hold-out test data (*n* = 114).

Algorithm	Model Performance			Prediction Performance		
	r	MAE	Weighted MAE	r	MAE	Weighted MAE
ARD	0.8268	7.4790	0.1133	0.7998	8.0453	0.1219
Lasso LAR	0.8290	7.4126	0.1123	0.7981	8.0473	0.1219
Lasso	0.8290	7.4129	0.1123	0.7981	8.0477	0.1219
MLP	0.7939	8.1039	0.1228	0.7779	8.0675	0.1222
PAR	0.8171	7.8135	0.1184	0.7902	8.2368	0.1248
XGBoost	0.8160	7.7096	0.1168	0.7918	8.2664	0.1252
Bayesian Ridge	0.8308	7.4376	0.1127	0.7945	8.2785	0.1254
GBM	0.8161	7.5873	0.1150	0.7818	8.3159	0.1260
Elastic Net	0.8343	7.3865	0.1119	0.7947	8.3217	0.1261
SVR	0.8303	7.5350	0.1142	0.7904	8.3845	0.1270
Ridge	0.8329	7.4285	0.1126	0.7934	8.3912	0.1271
GPR	0.7866	8.4452	0.1280	0.7719	8.3925	0.1272
LAR	0.8132	7.7176	0.1169	0.7837	8.4347	0.1278
LR	0.7832	8.5274	0.1292	0.7692	8.4450	0.1280
Huber	0.7966	8.1947	0.1242	0.7704	8.5157	0.1290
CatBoost	0.8299	7.6574	0.1160	0.7918	8.6085	0.1304
Theil–Sen	0.7862	8.4097	0.1274	0.7534	8.6277	0.1307
RVR	0.8322	7.4849	0.1134	0.7766	8.6291	0.1307
OMP	0.8029	7.9480	0.1204	0.7603	8.8267	0.1337
LightGBM	0.8196	7.6084	0.1153	0.7475	8.8588	0.1342
Extra Trees	0.8257	7.7683	0.1177	0.7876	8.9449	0.1355
Random Forest	0.8118	7.9223	0.1200	0.7679	8.9912	0.1362
Kernel Ridge	0.8316	7.5138	0.1138	0.7230	9.0415	0.1370
RANSAC	0.7772	8.655	0.1311	0.7384	9.1059	0.1380
AdaBoost	0.8211	7.7603	0.1176	0.7402	9.2366	0.1399
kNN	0.7769	8.3113	0.1259	0.7027	9.2521	0.1402
Decision Tree	0.7066	9.3118	0.1411	0.6315	9.8640	0.1495

Lasso = Least Absolute Shrinkage and Selection Operator; Lasso LAR = Lasso Least Angle Regression; SVR = Support Vector Regression; LAR = Least Angle Regression; Elastic Net = Elastic Net Regression; Bayesian Ridge = Bayesian Ridge Regression; Ridge = Ridge Regression; ARD = Automatic Relevance Determination; Random Forest = Random Forest Regression; PAR = Passive Aggressive Regression; CatBoost = Category Boosting Regression; RVR = Relevance Vector Regression; LightGBM = Light Gradient Boosting Machine; GBM = Gradient Boosting Machine; kNN = K-Nearest Neighbors; AdaBoost = Adaptive Boosting Regression; Extra Trees = Extra Trees Regression; XGBoost = Extreme Gradient Boosting; Kernel Ridge = Kernel Ridge Regression; GPR = Gaussian Processes Regression; MLP = Multi-layer Perceptron Regression; OMP = Orthogonal Matching Pursuit; LR = Linear Regression; Huber = Huber Regression; Theil–Sen = Theil–Sen Regression; RANSAC = Random Sample Consensus; Decision Tree = Decision Tree Regression.

**Table 4:** Comparison of computational speed of the algorithms for model training.

Algorithm	Training Time (s)			Average (SD) Training Time (s)
	HCP (*n* = 223)	Cam-CAN (*n* = 101)	IXI (*n* = 114)	
Automatic Relevance Determination	2.22	1.78	2.28	2.09 (0.27)
Bayesian Ridge Regression	0.79	0.77	0.75	0.77 (0.02)
Elastic Net Regression	1.09	0.34	0.15	0.53 (0.50)
Huber Regression	0.50	0.25	0.33	0.36 (0.13)
Least Angle Regression	0.18	0.07	0.13	0.13 (0.04)
Lasso Regression	0.55	0.34	0.22	0.37 (0.17)
Lasso Least Angle Regression	0.17	0.24	0.16	0.19 (0.04)
Linear Regression	0.61	0.58	0.55	0.58 (0.03)
Orthogonal Matching Pursuit	0.08	0.08	0.06	0.07 (0.01)
Passive Aggressive Regression	0.19	0.18	0.15	0.17 (0.02)
Random Sample Consensus	1.11	1.10	1.07	1.09 (0.02)
Ridge Regression	0.07	0.06	0.06	0.06 (0.01)
Relevance Vector Regression	4.87	3.56	2.25	3.56 (1.31)
Support Vector Regression	0.71	0.29	0.21	0.40 (0.27)
Theil-Sen Regression	58.41	58.84	57.67	58.31 (0.59)
Adaptive Boosting Regression	29.57	7.95	14.28	17.27 (11.12)
Category Boosting Regression	45.67	44.58	47.67	45.97 (1.28)
Decision Tree Regression	0.07	0.16	1.31	0.51 (0.69)
Extra Trees Regression	7.19	9.07	9.16	8.47 (1.11)
Gradient Boosting Machine	4.42	2.58	8.50	5.17 (3.03)
Light Gradient Boosting Machine	0.73	0.62	0.68	0.68 (0.06)
Random Forest Regression	5.49	7.43	9.18	7.37 (1.85)
Extreme Gradient Boosting	3.59	1.09	4.70	3.12 (1.51)
Gaussian Process Regression	0.82	0.31	0.26	0.46 (0.31)
K-Nearest Neighbors Regression	0.49	0.31	0.32	0.37 (0.10)
Kernel Ridge Regression	0.23	0.11	0.10	0.15 (0.07)
Multi-layer Perceptron Regression	3.91	4.94	6.08	4.98 (1.09)

## Data Availability

All data used in this study are publicly available and can be accessed directly from the Human Connectome Project (https://www.humanconnectome.org/study/hcp-young-adult, accessed on 1 September 2020), the Cambridge Centre for Ageing and Neuroscience (https://www.cam-can.org, accessed on 1 September 2020), and the Information eXtration from Images (https://brain-development.org, accessed on 1 September 2020) websites.

## Supplementary Materials

The following supporting information can be downloaded at: https://www.mdpi.com/article/10.3390/s22208077/s1, Figure S1: Definition of the Desikan-Killiany atlas, Figure S2: Scatter plots showing pairwise correlations between chronological age and predicted brain age across 27 algorithms for the HCP cohort, Figure S3: Scatter plots showing pairwise correlations between chronological age and predicted brain age across 27 algorithms for the Cam-CAN cohort, Figure S4: Scatter plots showing pairwise correlations between chronological age and predicted brain age across 27 algorithms for the IXI cohort, Figure S5: Scatter plots showing correlations of mean absolute SHAP values (feature importance) between models (Lasso, GPR, and GBM), Figure S6: Average accuracies of different feature dimensionality in the test sets for each algorithm (Lasso, GPR, and GBM) in the HCP, Cam-CAN, and IXI, Table S1: List of the anatomical regions of the Desikan-Killiany atlas, Table S2: Brain morphometric characteristics for the three cohorts, Table S3: Sample and demographic information for the three cohorts used for brain age prediction, Table S4: List of the top 20 regional features by mean absolute SHAP value for the HCP cohort, Table S5: List of the top 20 regional features by mean absolute SHAP value for the Cam-CAN cohort, Table S6: List of the top 20 regional features by mean absolute SHAP value for the IXI cohort. Table S7: Algorithm performance based on the structural features from the HCP individuals entered in the model for model performance in the training data (*n* = 890) and prediction performance in the hold-out test data (*n* = 223), Table S8: Algorithm performance based on the structural features from the Cam-CAN individuals entered in the model for model performance in the training data (*n* = 500) and prediction performance in the hold-out test data (*n* = 101), Table S9: Algorithm performance based on the structural features from the IXI individuals entered in the model for model performance in the training data (*n* = 453) and prediction performance in the hold-out test data (*n* = 114).
