# Supplementary material for: Brain Age Prediction: A Comparison between Machine Learning Models Using Brain Morphometric Data
Source: Sensors (Basel). 2022 Oct 21;22(20):8077. doi: 10.3390/s22208077 (PMC9608785; doi:10.3390/s22208077)
Supplement: Supplementary file 1 [file sensors-22-08077-s001.zip › sensors-1905269-supplementary.pdf]

Brain Age Prediction: A Comparison Between Machine Learning Models Using Brain Morphometric Data

Supplementary Materials

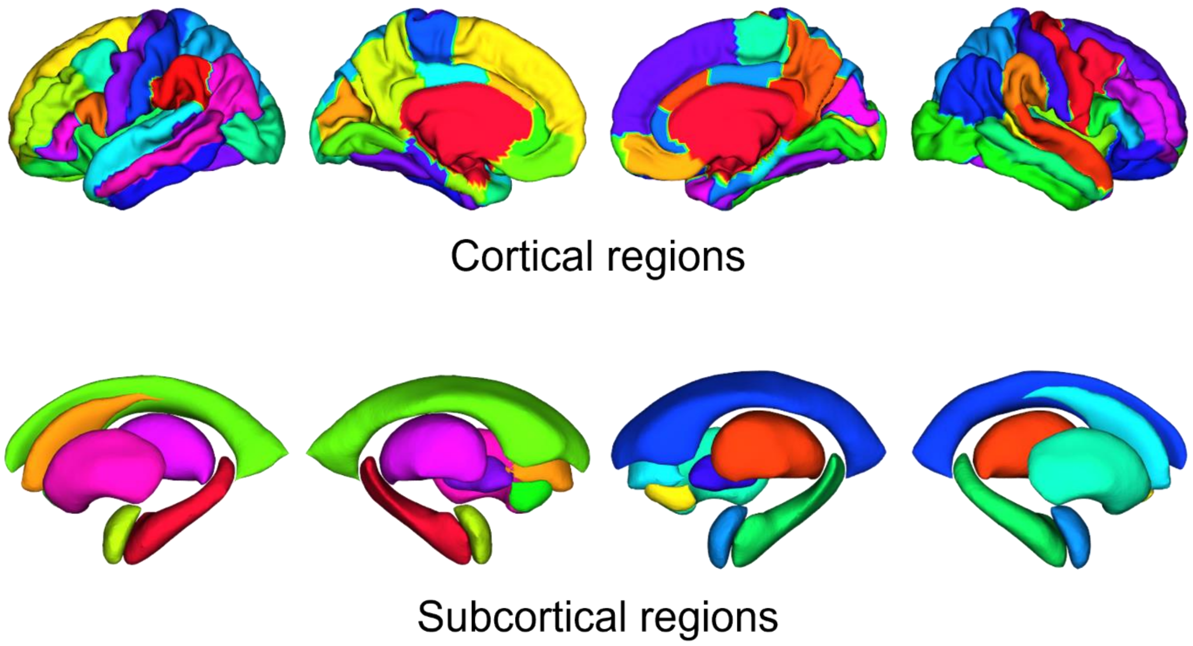

Supplementary Figure S1. Definition of the Desikan-Killiany atlas.

Supplementary Table S1. List of the anatomical regions of the Desikan-Killiany atlas

**Cortical regions**				
**Label number**	**Label Name**	**Hemisphere**	**Name**	**Lobe**
1	lh_bankssts	L	Banks of superior temporal sulcus	
2	lh_caudalanteriorcingulate	L	Caudal anterior cingulate cortex	Frontal
3	lh_caudalmiddlefrontal	L	Caudal middle frontal gyrus	Frontal
4	lh_cuneus	L	Cuneus	Occipital
5	lh_entorhinal	L	Entorhinal cortex	Temporal
6	lh_fusiform	L	Fusiform gyrus	Temporal
7	lh_inferiorparietal	L	Inferior parietal lobule	Parietal
8	lh_inferiortemporal	L	Inferior temporal gyrus	Temporal
9	lh_lateraloccipital	L	Lateral occipital gyrus	Occipital
10	lh_lateralorbitofrontal	L	Lateral orbitofrontal gyrus	Frontal
11	lh_lingual	L	Lingual gyrus	Occipital
12	lh_medialorbitofrontal	L	Medial orbitofrontal gyrus	Frontal
13	lh_middletemporal	L	Middle temporal gyrus	Temporal
14	lh_parahippocampal	L	Parahippocampal gyrus	Temporal
15	lh_paracentral	L	Paracentral gyrus	Frontal
16	lh_parsopercularis	L	Pars opercularis	Frontal
17	lh_parsorbitalis	L	Pars orbitalis	Frontal
18	lh_parstriangularis	L	Pars triangularis	Frontal
19	lh_pericalcarine	L	Pericalcarine gyrus	Occipital
20	lh_postcentral	L	Postcentral gyrus	Parietal
21	lh_posteriorcingulate	L	Posterior cingulate cortex	Parietal
22	lh_precentral	L	Precentral gyrus	Frontal
23	lh_precuneus	L	Precuneus	Parietal
24	lh_rostralanteriorcingulate	L	Rostral anterior cingulate cortex	Frontal
25	lh_rostralmiddlefrontal	L	Rostral middle frontal gyrus	Frontal
26	lh_superiorfrontal	L	Superior frontal gyrus	Frontal
27	lh_superiorparietal	L	Superior parietal lobule	Parietal
28	lh_superiortemporal	L	Superior temporal gyrus	Temporal
29	lh_supramarginal	L	Supramarginal gyrus	Parietal
30	lh_frontalpole	L	Frontal pole	Frontal
31	lh_temporalpole	L	Temporal pole	Temporal
32	lh_transversetemporal	L	Transverse temporal gyrus	Temporal
33	lh_insula	L	Insula	
34	lh_isthmuscingulate	L	Isthmus cingulate	
35	rh_bankssts	R	Banks of superior temporal sulcus	
36	rh_caudalanteriorcingulate	R	Caudal anterior cingulate cortex	Frontal
37	rh_caudalmiddlefrontal	R	Caudal middle frontal gyrus	Frontal
38	rh_cuneus	R	Cuneus	Occipital

39	rh_entorhinal	R	Entorhinal cortex	Temporal
40	rh_fusiform	R	Fusiform gyrus	Temporal
41	rh_inferiorparietal	R	Inferior parietal lobule	Parietal
42	rh_inferiortemporal	R	Inferior temporal gyrus	Temporal
43	rh_lateraloccipital	R	Lateral occipital gyrus	Occipital
44	rh_lateralorbitofrontal	R	Lateral orbitofrontal gyrus	Frontal
45	rh_lingual	R	Lingual gyrus	Occipital
46	rh_medialorbitofrontal	R	Medial orbitofrontal gyrus	Frontal
47	rh_middletemporal	R	Middle temporal gyrus	Temporal
48	rh_parahippocampal	R	Parahippocampal gyrus	Temporal
49	rh_paracentral	R	Paracentral gyrus	Frontal
50	rh_parsopercularis	R	Pars opercularis	Frontal
51	rh_parsorbitalis	R	Pars orbitalis	Frontal
52	rh_parstriangularis	R	Pars triangularis	Frontal
53	rh_pericalcarine	R	Pericalcarine gyrus	Occipital
54	rh_postcentral	R	Postcentral gyrus	Parietal
55	rh_posteriorcingulate	R	Posterior cingulate cortex	Parietal
56	rh_precentral	R	Precentral gyrus	Frontal
57	rh_precuneus	R	Precuneus	Parietal
58	rh_rostralanteriorcingulate	R	Rostral anterior cingulate cortex	Frontal
59	rh_rostralmiddlefrontal	R	Rostral middle frontal gyrus	Frontal
60	rh_superiorfrontal	R	Superior frontal gyrus	Frontal
61	rh_superiorparietal	R	Superior parietal lobule	Parietal
62	rh_superiortemporal	R	Superior temporal gyrus	Temporal
63	rh_supramarginal	R	Supramarginal gyrus	Parietal
64	rh_frontalpole	R	Frontal pole	Frontal
65	rh_temporalpole	R	Temporal pole	Temporal
66	rh_transversetemporal	R	Transverse temporal gyrus	Temporal
67	rh_insula	R	Insula	
68	rh_isthmuscingulate	R	Isthmus cingulate	
**Subcortical regions**				
69	lh_thalamus	L	Thalamus	
70	lh_hippocampus	L	Hippocampus	
71	lh_caudate	L	Caudate nucleus	
72	lh_accumbens	L	Nucleus Accumbens	
73	lh_pallidum	L	Pallidum	
74	lh_putamen	L	Putamen	
75	lh_amygdala	L	Amygdala	
76	lh_lateral_ventricle	L	Lateral ventricles	
77	rh_thalamus	R	Thalamus	
78	rh_hippocampus	R	Hippocampus	
79	rh_caudate	R	Caudate nucleus	
80	rh_accumbens	R	Nucleus Accumbens	
81	rh_pallidum	R	Pallidum	

82	rh_putamen	R	Putamen	
83	rh_amygdala	R	Amygdala	
84	rh_lateral_ventricle	R	Lateral ventricles	

Supplementary Table S2. Brain morphometric characteristics for the three cohorts

Label Name	HCP	Cam-CAN	IXI
	Mean ± SD (min, max)	Mean ± SD (min, max)	Mean ± SD (min, max)
**Cortical thickness**			
lh_bankssts	2.68 ± 0.14 (1.82, 3.04)	2.57 ± 0.17 (1.31, 3.05)	2.42 ± 0.19 (1.4, 2.9)
lh_caudalanteriorcingulate	2.69 ± 0.17 (2.0, 3.25)	2.66 ± 0.24 (1.5, 3.42)	2.41 ± 0.2 (1.63, 2.98)
lh_caudalmiddlefrontal	2.73 ± 0.12 (1.83, 3.15)	2.63 ± 0.17 (1.66, 3.12)	2.5 ± 0.14 (1.79, 2.87)
lh_cuneus	2.08 ± 0.11 (1.38, 2.45)	1.89 ± 0.16 (1.15, 2.44)	1.71 ± 0.15 (1.22, 2.23)
lh_entorhinal	3.3 ± 0.24 (2.28, 4.32)	3.44 ± 0.28 (2.08, 4.2)	3.16 ± 0.27 (1.95, 3.98)
lh_fusiform	2.88 ± 0.12 (1.85, 3.29)	2.8 ± 0.14 (1.51, 3.2)	2.55 ± 0.17 (1.61, 2.92)
lh_inferiorparietal	2.58 ± 0.11 (1.83, 2.97)	2.51 ± 0.15 (1.35, 2.95)	2.36 ± 0.13 (1.65, 2.66)
lh_inferiortemporal	2.94 ± 0.13 (2.03, 3.31)	2.89 ± 0.14 (1.9, 3.29)	2.7 ± 0.15 (2.06, 3.19)
lh_lateraloccipital	2.29 ± 0.11 (1.53, 2.56)	2.26 ± 0.13 (1.63, 2.66)	2.04 ± 0.12 (1.46, 2.41)
lh_lateralorbitofrontal	2.81 ± 0.12 (1.89, 3.33)	2.7 ± 0.14 (1.7, 3.15)	2.62 ± 0.18 (1.81, 3.3)
lh_lingual	2.17 ± 0.11 (1.36, 2.49)	2.03 ± 0.14 (1.39, 2.49)	1.78 ± 0.13 (1.36, 2.3)
lh_medialorbitofrontal	2.57 ± 0.15 (1.8, 3.18)	2.46 ± 0.14 (1.58, 3.06)	2.34 ± 0.19 (1.75, 3.05)
lh_middletemporal	2.98 ± 0.13 (2.05, 3.36)	2.92 ± 0.16 (1.77, 3.38)	2.73 ± 0.15 (1.94, 3.11)
lh_parahippocampal	2.72 ± 0.26 (1.78, 3.48)	2.84 ± 0.29 (1.73, 3.58)	2.65 ± 0.27 (1.75, 3.55)
lh_paracentral	2.55 ± 0.13 (1.66, 3.01)	2.44 ± 0.17 (1.18, 2.9)	2.24 ± 0.19 (1.36, 2.76)
lh_parsopercularis	2.78 ± 0.12 (1.9, 3.25)	2.62 ± 0.16 (1.54, 3.17)	2.52 ± 0.16 (1.65, 2.96)
lh_parsorbitalis	2.78 ± 0.15 (1.9, 3.2)	2.74 ± 0.19 (2.12, 3.24)	2.67 ± 0.21 (1.61, 3.45)
lh_parstriangularis	2.63 ± 0.13 (1.78, 3.17)	2.48 ± 0.16 (1.82, 3.0)	2.4 ± 0.16 (1.46, 2.95)
lh_pericalcarine	2.0 ± 0.12 (1.37, 2.45)	1.63 ± 0.14 (1.3, 2.13)	1.43 ± 0.13 (1.01, 2.02)
lh_postcentral	2.21 ± 0.1 (1.56, 2.55)	2.14 ± 0.15 (1.59, 2.75)	2.02 ± 0.13 (1.5, 2.48)
lh_posteriorcingulate	2.56 ± 0.13 (1.43, 2.92)	2.47 ± 0.16 (1.42, 2.93)	2.34 ± 0.17 (1.51, 2.88)
lh_precentral	2.73 ± 0.11 (1.78, 3.13)	2.63 ± 0.19 (1.77, 3.12)	2.43 ± 0.19 (1.49, 2.87)
lh_precuneus	2.52 ± 0.11 (1.74, 2.82)	2.42 ± 0.15 (1.16, 2.87)	2.26 ± 0.16 (1.34, 2.71)
lh_rostralanteriorcingulate	3.03 ± 0.18 (2.19, 3.63)	2.82 ± 0.2 (1.46, 3.55)	2.64 ± 0.2 (1.62, 3.35)
lh_rostralmiddlefrontal	2.57 ± 0.12 (1.9, 3.01)	2.43 ± 0.13 (1.71, 2.81)	2.35 ± 0.12 (1.77, 2.63)

lh_superiorfrontal	2.84 ± 0.13 (2.05, 3.26)	2.74 ± 0.17 (1.65, 3.19)	2.68 ± 0.15 (1.89, 3.12)
lh_superiorparietal	2.29 ± 0.1 (1.62, 2.55)	2.27 ± 0.14 (1.68, 2.7)	2.12 ± 0.11 (1.65, 2.43)
lh_superiortemporal	2.89 ± 0.13 (2.08, 3.3)	2.82 ± 0.18 (1.73, 3.41)	2.67 ± 0.16 (1.8, 3.05)
lh_supramarginal	2.65 ± 0.11 (1.89, 3.03)	2.6 ± 0.16 (1.51, 3.09)	2.47 ± 0.14 (1.79, 2.81)
lh_frontalpole	2.84 ± 0.2 (1.94, 3.65)	2.81 ± 0.23 (2.12, 3.82)	2.73 ± 0.23 (1.76, 3.42)
lh_temporalpole	3.42 ± 0.27 (2.56, 4.26)	3.63 ± 0.28 (2.16, 4.37)	3.57 ± 0.31 (2.09, 4.26)
lh_transversetemporal	2.66 ± 0.17 (1.6, 3.17)	2.45 ± 0.24 (1.3, 3.18)	2.24 ± 0.24 (1.3, 2.84)
lh_insula	3.06 ± 0.15 (2.13, 3.46)	2.95 ± 0.18 (1.64, 3.37)	2.84 ± 0.19 (1.56, 3.41)
lh_isthmuscingulate	2.28 ± 0.3 (0.95, 2.8)	2.34 ± 0.19 (1.72, 2.95)	2.23 ± 0.19 (1.61, 2.94)
rh_bankssts	2.79 ± 0.14 (2.24, 3.25)	2.65 ± 0.18 (1.22, 3.09)	2.49 ± 0.19 (1.56, 2.98)
rh_caudalanteriorcingulate	2.52 ± 0.23 (1.32, 3.13)	2.49 ± 0.2 (1.4, 3.09)	2.34 ± 0.18 (1.77, 2.91)
rh_caudalmiddlefrontal	2.75 ± 0.12 (2.36, 3.1)	2.58 ± 0.17 (1.57, 3.13)	2.47 ± 0.15 (1.78, 2.92)
rh_cuneus	2.09 ± 0.11 (1.67, 2.45)	1.96 ± 0.15 (1.32, 2.47)	1.72 ± 0.13 (1.26, 2.16)
rh_entorhinal	3.42 ± 0.25 (2.66, 4.22)	3.5 ± 0.33 (1.9, 4.5)	3.18 ± 0.31 (1.68, 3.99)
rh_fusiform	2.9 ± 0.11 (2.49, 3.27)	2.81 ± 0.15 (1.57, 3.21)	2.55 ± 0.18 (1.57, 3.02)
rh_inferiorparietal	2.65 ± 0.1 (2.23, 2.99)	2.53 ± 0.15 (1.36, 2.96)	2.38 ± 0.13 (1.7, 2.68)
rh_inferiortemporal	2.97 ± 0.12 (2.56, 3.31)	2.87 ± 0.14 (1.95, 3.35)	2.7 ± 0.16 (1.89, 3.27)
rh_lateraloccipital	2.34 ± 0.11 (2.0, 2.71)	2.33 ± 0.14 (1.46, 2.88)	2.09 ± 0.13 (1.54, 2.42)
rh_lateralorbitofrontal	2.83 ± 0.12 (2.29, 3.22)	2.7 ± 0.15 (1.64, 3.16)	2.58 ± 0.2 (1.87, 3.49)
rh_lingual	2.19 ± 0.12 (1.77, 2.57)	2.08 ± 0.13 (1.31, 2.46)	1.8 ± 0.13 (1.32, 2.4)
rh_medialorbitofrontal	2.74 ± 0.14 (2.29, 3.29)	2.47 ± 0.14 (1.63, 3.06)	2.38 ± 0.17 (1.71, 2.95)
rh_middletemporal	3.05 ± 0.12 (2.59, 3.41)	2.94 ± 0.15 (1.74, 3.41)	2.77 ± 0.15 (1.73, 3.18)
rh_parahippocampal	2.7 ± 0.21 (2.0, 3.41)	2.81 ± 0.25 (1.84, 3.46)	2.57 ± 0.23 (1.77, 3.2)
rh_paracentral	2.58 ± 0.13 (2.14, 3.0)	2.48 ± 0.16 (1.43, 2.94)	2.24 ± 0.19 (1.32, 2.83)
rh_parsopercularis	2.82 ± 0.12 (2.38, 3.25)	2.61 ± 0.17 (1.32, 3.21)	2.52 ± 0.18 (1.67, 3.01)
rh_parsorbitalis	2.83 ± 0.15 (1.71, 3.5)	2.75 ± 0.18 (1.87, 3.42)	2.68 ± 0.21 (1.52, 3.34)
rh_parstriangularis	2.7 ± 0.12 (2.27, 3.2)	2.47 ± 0.17 (1.52, 3.06)	2.43 ± 0.16 (1.78, 2.81)
rh_pericalcarine	2.0 ± 0.12 (1.58, 2.4)	1.66 ± 0.15 (1.11, 2.14)	1.43 ± 0.12 (1.04, 1.87)
rh_postcentral	2.24 ± 0.1 (1.84, 2.64)	2.11 ± 0.15 (1.55, 2.73)	2.0 ± 0.13 (1.5, 2.52)

rh_posteriorcingulate	2.53 ± 0.15<br>(1.71, 2.96)	2.44 ± 0.17<br>(1.5, 2.98)	2.37 ± 0.17<br>(1.48, 2.94)
rh_precentral	2.73 ± 0.11<br>(2.19, 3.11)	2.57 ± 0.19<br>(1.55, 3.0)	2.41 ± 0.18<br>(1.56, 2.84)
rh_precuneus	2.55 ± 0.11<br>(2.15, 2.88)	2.43 ± 0.15<br>(1.39, 2.83)	2.26 ± 0.15<br>(1.41, 2.7)
rh_rostralanteriorcingulate	3.01 ± 0.2<br>(2.37, 3.74)	2.84 ± 0.21<br>(1.42, 3.74)	2.69 ± 0.19<br>(1.91, 3.4)
rh_rostralmiddlefrontal	2.59 ± 0.12<br>(2.12, 2.96)	2.37 ± 0.12<br>(1.7, 2.81)	2.33 ± 0.12<br>(1.85, 2.64)
rh_superiorfrontal	2.87 ± 0.13<br>(2.41, 3.29)	2.71 ± 0.16<br>(1.69, 3.15)	2.68 ± 0.15<br>(1.83, 3.13)
rh_superiorparietal	2.32 ± 0.1<br>(1.93, 2.62)	2.25 ± 0.15<br>(1.72, 2.72)	2.11 ± 0.12<br>(1.67, 2.43)
rh_superiortemporal	2.94 ± 0.13<br>(2.49, 3.34)	2.84 ± 0.18<br>(1.73, 3.29)	2.73 ± 0.17<br>(1.81, 3.16)
rh_supramarginal	2.7 ± 0.11<br>(2.3, 3.02)	2.6 ± 0.17<br>(1.34, 3.01)	2.46 ± 0.14<br>(1.8, 2.86)
rh_frontalpole	2.86 ± 0.2<br>(1.9, 3.68)	2.77 ± 0.24<br>(2.14, 3.82)	2.71 ± 0.24<br>(2.0, 3.63)
rh_temporalpole	3.65 ± 0.3<br>(2.43, 4.46)	3.72 ± 0.31<br>(2.22, 4.51)	3.65 ± 0.31<br>(1.95, 4.34)
rh_transversetemporal	2.74 ± 0.17<br>(2.18, 3.39)	2.44 ± 0.24<br>(1.42, 3.22)	2.29 ± 0.27<br>(1.29, 3.0)
rh_insula	3.01 ± 0.14<br>(2.5, 3.44)	3.0 ± 0.19<br>(1.58, 3.52)	2.87 ± 0.2<br>(1.67, 3.42)
rh_isthmuscingulate	2.32 ± 0.17<br>(1.3, 2.86)	2.36 ± 0.2<br>(1.36, 3.02)	2.25 ± 0.18<br>(1.56, 2.78)
**Surface area**			
lh_bankssts	1053.38 ± 171.64<br>(665.0, 1846.0)	986.34 ± 165.16<br>(548.0, 1648.0)	976.84 ± 180.32<br>(594.0, 1980.0)
lh_caudalanteriorcingulate	691.34 ± 147.15<br>(371.0, 1271.0)	622.14 ± 137.16<br>(242.0, 1093.0)	602.71 ± 155.23<br>(244.0, 1165.0)
lh_caudalmiddlefrontal	2396.83 ± 413.25<br>(1405.0, 4114.0)	2239.66 ± 353.42<br>(1389.0, 3484.0)	2136.89 ± 349.59<br>(1320.0, 3369.0)
lh_cuneus	1460.6 ± 211.81<br>(832.0, 2241.0)	1516.21 ± 227.26<br>(780.0, 2294.0)	1493.34 ± 235.08<br>(922.0, 2578.0)
lh_entorhinal	427.6 ± 82.98<br>(264.0, 827.0)	456.67 ± 94.27<br>(209.0, 788.0)	425.52 ± 91.1<br>(220.0, 769.0)
lh_fusiform	3388.54 ± 469.68<br>(2335.0, 5810.0)	3084.33 ± 413.82<br>(2095.0, 4423.0)	3083.27 ± 428.87<br>(1998.0, 4527.0)
lh_inferiorparietal	4673.49 ± 688.65<br>(2917.0, 6879.0)	4332.76 ± 638.5<br>(2651.0, 6728.0)	4259.61 ± 654.19<br>(2795.0, 6664.0)
lh_inferiortemporal	3416.37 ± 508.99<br>(2179.0, 5703.0)	3291.04 ± 516.25<br>(1917.0, 5329.0)	3354.84 ± 549.25<br>(1905.0, 5180.0)
lh_lateraloccipital	4714.47 ± 608.92<br>(3197.0, 6860.0)	4961.33 ± 682.67<br>(3466.0, 7890.0)	4917.11 ± 698.71<br>(3165.0, 11152.0)
lh_lateralorbitofrontal	2651.36 ± 312.34<br>(1706.0, 3672.0)	2604.8 ± 306.53<br>(1835.0, 3637.0)	2601.02 ± 387.44<br>(1379.0, 3863.0)
lh_lingual	3100.65 ± 438.81<br>(1876.0, 4880.0)	2974.76 ± 391.85<br>(2013.0, 4251.0)	2988.85 ± 440.46<br>(1873.0, 5771.0)
lh_medialorbitofrontal	1979.43 ± 300.2<br>(1239.0, 2980.0)	1877.2 ± 236.07<br>(1249.0, 2842.0)	1905.66 ± 257.9<br>(1214.0, 2680.0)
lh_middletemporal	3158.1 ± 430.01<br>(1979.0, 4750.0)	3104.57 ± 453.27<br>(2011.0, 5027.0)	3144.52 ± 477.53<br>(2043.0, 4879.0)
lh_parahippocampal	732.64 ± 117.89<br>(435.0, 1495.0)	651.31 ± 84.01<br>(449.0, 1136.0)	641.27 ± 80.7<br>(435.0, 967.0)

lh_paracentral	1316.36 ± 187.07<br>(856.0, 2068.0)	1372.41 ± 160.49<br>(994.0, 1950.0)	1301.99 ± 174.4<br>(868.0, 2022.0)
lh_parsopercularis	1708.95 ± 277.34<br>(1128.0, 2868.0)	1551.55 ± 246.14<br>(1005.0, 2606.0)	1541.88 ± 258.81<br>(977.0, 2832.0)
lh_parsorbitalis	637.36 ± 86.02<br>(399.0, 902.0)	683.42 ± 98.47<br>(433.0, 1046.0)	684.83 ± 105.24<br>(350.0, 1024.0)
lh_parstriangularis	1305.33 ± 203.1<br>(865.0, 2098.0)	1295.01 ± 207.3<br>(878.0, 2058.0)	1285.38 ± 223.94<br>(818.0, 2059.0)
lh_pericalcarine	1476.24 ± 244.24<br>(733.0, 2203.0)	1385.14 ± 244.49<br>(805.0, 2302.0)	1348.61 ± 263.14<br>(736.0, 2983.0)
lh_postcentral	4152.85 ± 504.64<br>(2872.0, 6136.0)	4072.61 ± 480.65<br>(2901.0, 5627.0)	3953.65 ± 530.9<br>(2047.0, 6302.0)
lh_posteriorcingulate	1223.09 ± 191.39<br>(794.0, 2742.0)	1168.6 ± 187.72<br>(508.0, 1842.0)	1083.22 ± 199.62<br>(516.0, 1848.0)
lh_precentral	4817.05 ± 558.52<br>(3547.0, 8794.0)	4835.93 ± 531.06<br>(3424.0, 7071.0)	4637.6 ± 533.64<br>(2921.0, 6464.0)
lh_precuneus	3860.43 ± 504.58<br>(2554.0, 5685.0)	3775.1 ± 509.66<br>(2608.0, 5858.0)	3644.35 ± 545.93<br>(2185.0, 5573.0)
lh_rostralanteriorcingulate	873.77 ± 166.85<br>(441.0, 1457.0)	836.8 ± 167.49<br>(420.0, 1756.0)	821.56 ± 180.75<br>(301.0, 1350.0)
lh_rostralmiddlefrontal	6006.56 ± 785.81<br>(4049.0, 8461.0)	5567.62 ± 815.46<br>(3606.0, 8699.0)	5471.95 ± 847.7<br>(3253.0, 8234.0)
lh_superiorfrontal	7367.41 ± 909.43<br>(5087.0, 10695.0)	7344.35 ± 949.15<br>(5085.0, 11235.0)	7098.37 ± 917.23<br>(5129.0, 9978.0)
lh_superiorparietal	5495.69 ± 688.6<br>(3608.0, 8020.0)	5301.95 ± 708.18<br>(3672.0, 7965.0)	5158.11 ± 707.39<br>(2768.0, 7357.0)
lh_superiortemporal	3838.03 ± 467.85<br>(2622.0, 5381.0)	3875.5 ± 475.41<br>(2606.0, 5786.0)	3897.28 ± 511.27<br>(2606.0, 5867.0)
lh_supramarginal	3950.25 ± 591.19<br>(2338.0, 6190.0)	3985.89 ± 677.89<br>(2345.0, 6548.0)	3939.01 ± 676.56<br>(2081.0, 6323.0)
lh_frontalpole	202.72 ± 35.61<br>(84.0, 317.0)	250.13 ± 30.87<br>(158.0, 367.0)	262.08 ± 34.46<br>(166.0, 368.0)
lh_temporalpole	499.32 ± 60.04<br>(331.0, 725.0)	470.71 ± 64.24<br>(291.0, 717.0)	484.05 ± 67.89<br>(255.0, 705.0)
lh_transversetemporal	451.43 ± 77.16<br>(285.0, 735.0)	445.14 ± 75.06<br>(269.0, 727.0)	448.0 ± 73.58<br>(262.0, 850.0)
lh_insula	2280.14 ± 277.95<br>(1587.0, 3778.0)	2420.41 ± 282.14<br>(1727.0, 3248.0)	2368.4 ± 306.48<br>(1530.0, 3460.0)
lh_isthmuscingulate	1123.58 ± 388.53<br>(641.0, 3189.0)	1011.94 ± 166.44<br>(613.0, 1559.0)	956.99 ± 164.76<br>(492.0, 1545.0)
rh_bankssts	976.6 ± 144.02<br>(609.0, 1568.0)	890.76 ± 131.25<br>(556.0, 1646.0)	871.41 ± 134.47<br>(506.0, 1346.0)
rh_caudalanteriorcingulate	828.71 ± 211.06<br>(411.0, 2079.0)	718.1 ± 153.08<br>(250.0, 1333.0)	695.25 ± 168.7<br>(213.0, 1368.0)
rh_caudalmiddlefrontal	2216.54 ± 399.24<br>(1176.0, 3666.0)	2158.12 ± 351.13<br>(1338.0, 3259.0)	2034.54 ± 346.82<br>(1216.0, 3443.0)
rh_cuneus	1500.53 ± 231.56<br>(536.0, 2292.0)	1615.99 ± 239.9<br>(1060.0, 2512.0)	1593.99 ± 239.71<br>(977.0, 2594.0)
rh_entorhinal	361.34 ± 84.01<br>(175.0, 720.0)	417.93 ± 85.47<br>(214.0, 796.0)	386.14 ± 79.83<br>(207.0, 776.0)
rh_fusiform	3291.5 ± 468.88<br>(2151.0, 5018.0)	3001.78 ± 412.6<br>(2016.0, 4400.0)	2987.5 ± 417.22<br>(1920.0, 4574.0)
rh_inferiorparietal	5619.08 ± 786.66<br>(3599.0, 8234.0)	5159.38 ± 762.51<br>(3366.0, 8206.0)	5101.41 ± 804.26<br>(3197.0, 8136.0)
rh_inferiortemporal	3272.24 ± 487.16<br>(1969.0, 4849.0)	3234.49 ± 486.02<br>(2077.0, 4837.0)	3186.9 ± 551.44<br>(1565.0, 5299.0)
rh_lateraloccipital	4612.24 ± 634.81<br>(3042.0, 6691.0)	4950.63 ± 691.67<br>(3383.0, 7858.0)	4946.86 ± 706.41<br>(3421.0, 9816.0)

rh_lateralorbitofrontal	2584.27 ± 320.3 (1647.0, 3859.0)	2529.25 ± 331.83 (1664.0, 3617.0)	2659.53 ± 386.17 (1518.0, 3886.0)
rh_lingual	3119.82 ± 430.54 (1955.0, 4921.0)	3168.98 ± 434.64 (2064.0, 4557.0)	3128.77 ± 444.36 (2058.0, 4578.0)
rh_medialorbitofrontal	1842.98 ± 228.0 (1161.0, 2886.0)	1943.4 ± 227.14 (1201.0, 2757.0)	2046.34 ± 255.02 (1234.0, 2906.0)
rh_middletemporal	3502.88 ± 465.15 (2259.0, 5225.0)	3439.19 ± 455.83 (2315.0, 5718.0)	3439.75 ± 501.62 (1993.0, 5186.0)
rh_parahippocampal	701.38 ± 106.96 (412.0, 1232.0)	628.2 ± 79.79 (423.0, 1050.0)	612.17 ± 82.92 (403.0, 960.0)
rh_paracentral	1508.87 ± 226.22 (1009.0, 2414.0)	1528.36 ± 193.67 (1033.0, 2442.0)	1457.07 ± 213.66 (887.0, 2450.0)
rh_parsopercularis	1439.96 ± 247.47 (859.0, 2864.0)	1326.63 ± 202.01 (873.0, 2492.0)	1299.66 ± 197.66 (823.0, 2243.0)
rh_parsorbitalis	793.96 ± 104.51 (485.0, 1121.0)	822.81 ± 112.62 (558.0, 1157.0)	828.62 ± 120.84 (455.0, 1234.0)
rh_parstriangularis	1523.83 ± 248.76 (913.0, 2393.0)	1492.01 ± 237.97 (944.0, 2350.0)	1480.25 ± 240.02 (918.0, 2259.0)
rh_pericalcarine	1614.86 ± 259.73 (726.0, 2484.0)	1554.12 ± 269.68 (967.0, 2378.0)	1500.53 ± 272.49 (923.0, 3045.0)
rh_postcentral	3974.74 ± 483.7 (2744.0, 6076.0)	3960.2 ± 460.72 (2622.0, 5779.0)	3845.02 ± 491.44 (1975.0, 5310.0)
rh_posteriorcingulate	1261.45 ± 215.38 (773.0, 2248.0)	1173.58 ± 188.33 (454.0, 1803.0)	1104.01 ± 217.61 (526.0, 2042.0)
rh_precentral	4896.35 ± 573.52 (3359.0, 7898.0)	4836.9 ± 530.77 (3518.0, 6821.0)	4659.18 ± 534.85 (3403.0, 6657.0)
rh_precuneus	4074.24 ± 560.2 (2576.0, 6051.0)	3945.72 ± 530.99 (2771.0, 6000.0)	3784.85 ± 547.35 (2471.0, 5847.0)
rh_rostralanteriorcingulate	674.04 ± 141.67 (321.0, 1134.0)	596.02 ± 129.23 (245.0, 1004.0)	599.97 ± 141.44 (226.0, 1374.0)
rh_rostralmiddlefrontal	6238.1 ± 819.03 (4311.0, 8773.0)	5779.82 ± 836.16 (3623.0, 8979.0)	5670.49 ± 877.3 (3384.0, 8120.0)
rh_superiorfrontal	7208.25 ± 900.78 (5014.0, 11507.0)	7066.97 ± 938.17 (4811.0, 10224.0)	6801.87 ± 938.02 (4409.0, 10145.0)
rh_superiorparietal	5501.73 ± 679.77 (3758.0, 7799.0)	5243.15 ± 655.57 (3197.0, 7605.0)	5086.26 ± 697.03 (2993.0, 7023.0)
rh_superiortemporal	3655.23 ± 427.22 (2622.0, 5245.0)	3623.12 ± 423.85 (2529.0, 5258.0)	3629.2 ± 430.03 (2321.0, 5190.0)
rh_supramarginal	3718.07 ± 550.78 (2207.0, 5570.0)	3638.1 ± 551.2 (2189.0, 5862.0)	3536.38 ± 540.26 (2063.0, 5519.0)
rh_frontalpole	279.6 ± 46.57 (145.0, 465.0)	303.73 ± 37.44 (185.0, 437.0)	325.84 ± 44.61 (221.0, 501.0)
rh_temporalpole	437.66 ± 57.47 (279.0, 642.0)	463.15 ± 61.7 (275.0, 716.0)	474.43 ± 65.51 (282.0, 685.0)
rh_transversetemporal	333.36 ± 55.6 (196.0, 545.0)	329.44 ± 50.5 (198.0, 533.0)	324.52 ± 49.72 (189.0, 503.0)
rh_insula	2416.25 ± 294.51 (1568.0, 3401.0)	2365.08 ± 305.29 (1662.0, 3690.0)	2255.7 ± 308.63 (1526.0, 3528.0)
rh_isthmuscingulate	986.03 ± 172.69 (597.0, 2045.0)	906.29 ± 142.14 (387.0, 1476.0)	857.27 ± 140.75 (500.0, 1365.0)
**Subcortical regions**			
lh_thalamus	8458.28 ± 917.31 (5945.0, 11399.0)	7379.46 ± 1016.95 (4773.9, 10102.6)	7887.47 ± 1133.83 (4862.6, 13008.3)
lh_hippocampus	4421.63 ± 486.12 (1262.0, 6037.0)	3935.66 ± 463.96 (2459.0, 5674.6)	4145.08 ± 492.97 (2948.8, 5587.3)
lh_caudate	3809.0 ± 476.3 (2575.0, 5546.0)	3411.67 ± 470.37 (2327.6, 5464.7)	3260.05 ± 463.68 (2141.9, 5324.4)

lh_accumbens	564.41 ± 93.32 (317.0, 1039.0)	347.55 ± 88.17 (131.2, 636.7)	402.64 ± 105.21 (144.2, 874.5)
lh_pallidum	1361.7 ± 235.78 (728.0, 2325.0)	2078.38 ± 274.19 (1423.8, 3255.8)	1986.89 ± 319.71 (1327.5, 4357.7)
lh_putamen	5519.14 ± 733.5 (3663.0, 7832.0)	4479.68 ± 609.13 (2452.8, 6548.0)	4504.39 ± 668.87 (2764.9, 6738.9)
lh_amygdala	1555.67 ± 204.41 (1029.0, 2307.0)	1524.7 ± 241.73 (913.7, 2308.6)	1490.89 ± 239.86 (373.5, 2223.3)
lh_lateral_ventricle	6477.73 ± 3612.59 (1512.0, 25798.0)	12952.04 ± 8919.29 (2513.0, 63928.2)	9530.91 ± 5730.23 (2251.3, 46817.4)
rh_thalamus	7395.51 ± 779.14 (5406.0, 10186.0)	7196.68 ± 926.59 (4670.9, 10040.7)	7268.21 ± 917.56 (4935.2, 11513.2)
rh_hippocampus	4487.01 ± 459.66 (2351.0, 6053.0)	4068.98 ± 483.31 (2737.3, 6151.1)	4240.42 ± 497.69 (2803.6, 5867.0)
rh_caudate	3929.9 ± 486.87 (2604.0, 5749.0)	3486.72 ± 486.67 (2229.4, 6055.6)	3355.72 ± 469.05 (2128.3, 5136.5)
rh_accumbens	599.66 ± 99.93 (324.0, 907.0)	473.93 ± 91.44 (218.7, 782.7)	502.15 ± 106.62 (262.9, 843.3)
rh_pallidum	1495.2 ± 200.54 (747.0, 2276.0)	2021.31 ± 271.32 (1368.2, 3016.9)	2021.71 ± 317.2 (1346.8, 3577.6)
rh_putamen	5582.43 ± 638.59 (3807.0, 8102.0)	4608.44 ± 624.23 (2668.7, 6643.6)	4606.17 ± 639.34 (3095.0, 6550.5)
rh_amygdala	1636.84 ± 217.59 (1000.0, 2847.0)	1688.84 ± 237.88 (786.8, 2595.5)	1690.71 ± 246.05 (938.3, 2906.9)
rh_lateral_ventricle	6003.53 ± 3243.89 (1188.0, 27206.0)	11712.6 ± 7863.29 (1644.8, 50952.1)	8804.38 ± 5501.47 (2272.7, 48357.2)

Supplementary Table S3. Sample and demographic information for the three cohorts used for brain age prediction

Cohort	Sample size	Males/Females	Age range (mean ± SD)
**Datasets used in the training set**			
**HCP**	890	403/487	28.79 ± 3.70
**Cam-CAN**	500	249/251	54.40 ± 18.26
**IXI**	453	200/253	48.13 ± 16.54
**Hold-out test datasets**			
**HCP**	223	104/119	28.85 ± 3.69
**Cam-CAN**	101	50/51	54.38 ± 17.51
**IXI**	114	51/63	48.85 ± 16.32

Supplementary Table S4. List of the top 20 regional features by mean absolute SHAP value for the HCP cohort.

**Least Absolute Shrinkage and Selection Operator (Lasso) Regression**	
**Feature**	**SHAP value**
Total intracranial volume	1.2464
Right inferior parietal lobule surface area	0.4976
Left pallidum volume	0.3981
Left superior frontal gyrus cortical thickness	0.3069
Right putamen volume	0.2946
Right middle temporal gyrus surface area	0.2569
Left inferior temporal gyrus cortical thickness	0.2536
Left lateral orbitofrontal gyrus surface area	0.2401
Left posterior cingulate cortex cortical thickness	0.2303
Left caudal middle frontal gyrus cortical thickness	0.2234
Left superior frontal gyrus surface area	0.2227
Left pericalcarine gyrus surface area	0.2013
Left pars triangularis cortical thickness	0.2001
Left middle temporal gyrus surface area	0.1948
Right frontal pole surface area	0.1914
Right insula cortical thickness	0.1860
Left lingual gyrus cortical thickness	0.1784
Right insula surface area	0.1770
Right hippocampus volume	0.1696
Left precuneus surface area	0.1618
**Gaussian Process Regression**	
**Feature**	**SHAP value**
Total intracranial volume	3.4492
Right inferior parietal lobule surface area	0.7329
Left superior frontal gyrus surface area	0.6324
Left superior frontal gyrus cortical thickness	0.6018
Left rostral middle frontal gyrus cortical thickness	0.5512
Left pallidum volume	0.4568
Right putamen volume	0.4552
Left pericalcarine gyrus surface area	0.4398
Left lateral orbitofrontal gyrus surface area	0.4149
Right thalamus volume	0.4047
Left caudal middle frontal gyrus cortical thickness	0.3955
Right middle temporal gyrus surface area	0.3938

Right superior temporal gyrus surface area	0.3915
Right supramarginal gyrus cortical thickness	0.3741
Left precentral gyrus cortical thickness	0.3660
Right postcentral gyrus cortical thickness	0.3525
Right lateral orbitofrontal gyrus cortical thickness	0.3474
Right lingual gyrus surface area	0.3265
Left lingual gyrus cortical thickness	0.3219
Left pericalcarine gyrus cortical thickness	0.3115
**Gradient Boosting Machine**	
**Feature**	**SHAP value**
Left pallidum volume	0.2522
Left caudal middle frontal gyrus cortical thickness	0.2170
Left posterior cingulate cortex cortical thickness	0.1630
Left middle temporal gyrus surface area	0.1283
Right superior frontal gyrus cortical thickness	0.1263
Left superior frontal gyrus cortical thickness	0.1257
Left temporal pole cortical thickness	0.1234
Left precuneus surface area	0.1231
Left insula cortical thickness	0.1216
Left lateral orbitofrontal gyrus cortical thickness	0.1146
Left isthmus cingulate surface area	0.1140
Left pars triangularis cortical thickness	0.1099
Total intracranial volume	0.1089
Left thalamus volume	0.1083
Right lateral orbitofrontal gyrus surface area	0.1075
Right insula surface area	0.1066
Right inferior parietal lobule surface area	0.1043
Right hippocampus volume	0.0993
Right precentral gyrus cortical thickness	0.0992
Right caudal middle frontal gyrus cortical thickness	0.0963

Supplementary Table S5. List of the top 20 regional features by mean absolute SHAP value for the Cam-CAN cohort.

**Least Absolute Shrinkage and Selection Operator (Lasso) Regression**	
**Feature**	**SHAP value**
Total intracranial volume	7.7143
Left thalamus volume	2.8638
Left superior frontal gyrus cortical thickness	2.2929
Left superior frontal gyrus surface area	1.5312
Right precentral gyrus surface area	1.3444
Left precuneus cortical thickness	1.3436
Left insula surface area	1.3076
Right precentral gyrus cortical thickness	1.2582
Left supramarginal gyrus cortical thickness	1.2476
Left amygdala volume	1.2339
Left fusiform gyrus surface area	1.1621
Left superior parietal lobule surface area	1.1137
Right caudal middle frontal gyrus cortical thickness	1.0820
Left superior temporal gyrus cortical thickness	1.0483
Left temporal pole cortical thickness	1.0472
Left lateral orbitofrontal gyrus surface area	0.9624
Left posterior cingulate cortex surface area	0.9527
Right lateral occipital gyrus surface area	0.9420
Right superior parietal lobule cortical thickness	0.8872
Left entorhinal cortex cortical thickness	0.8868
**Gaussian Process Regression**	
**Feature**	**SHAP value**
Total intracranial volume	8.8313
Left superior frontal gyrus cortical thickness	3.0479
Left thalamus volume	2.4525
Left precuneus cortical thickness	2.1928
Left pallidum volume	1.9247
Left superior frontal gyrus surface area	1.9213
Right precuneus cortical thickness	1.8973
Right precentral gyrus surface area	1.7306
Left amygdala volume	1.5316
Left supramarginal gyrus cortical thickness	1.4707
Left lateral orbitofrontal gyrus surface area	1.4277
Left pericalcarine gyrus surface area	1.3613

Right caudal middle frontal gyrus cortical thickness	1.3169
Left posterior cingulate cortex surface area	1.2871
Right superior temporal gyrus surface area	1.2820
Right cuneus surface area	1.2775
Right lateral occipital gyrus surface area	1.2592
Left superior parietal lobule surface area	1.2243
Right rostral middle frontal gyrus cortical thickness	1.2199
Right thalamus volume	1.2174
**Gradient Boosting Machine**	
**Feature**	**SHAP value**
Left lateral ventricles volume	1.0214
Right lateral ventricles volume	0.9673
Left thalamus volume	0.9094
Total intracranial volume	0.7257
Left superior frontal gyrus cortical thickness	0.7183
Right nucleus accumbens volume	0.6493
Right supramarginal gyrus cortical thickness	0.6390
Left superior temporal gyrus cortical thickness	0.6242
Right superior temporal gyrus cortical thickness	0.6190
Right precentral gyrus cortical thickness	0.5824
Left precentral gyrus cortical thickness	0.4496
Left supramarginal gyrus cortical thickness	0.4224
Right superior frontal gyrus cortical thickness	0.4029
Right pars opercularis cortical thickness	0.3645
Left amygdala volume	0.3140
Right putamen volume	0.2894
Left temporal pole cortical thickness	0.2730
Left precuneus cortical thickness	0.2694
Right inferior parietal lobule cortical thickness	0.2604
Right insula cortical thickness	0.2603

Supplementary Table S6. List of the top 20 regional features by mean absolute SHAP value for the IXI cohort.

**Least Absolute Shrinkage and Selection Operator (Lasso) Regression**	
**Feature**	**SHAP value**
Total intracranial volume	4.3064
Left superior frontal gyrus cortical thickness	2.8943
Left thalamus volume	2.3970
Right middle temporal gyrus surface area	2.3649
Left superior frontal gyrus surface area	2.0884
Right precuneus surface area	1.5263
Left pericalcarine gyrus surface area	1.4622
Right inferior parietal lobule cortical thickness	1.4438
Right cuneus surface area	1.3514
Left superior temporal gyrus surface area	1.2764
Right putamen volume	1.2621
Right temporal pole surface area	1.1248
Right precentral gyrus surface area	1.0995
Right pars triangularis cortical thickness	1.0666
Left lateral orbitofrontal gyrus surface area	0.9724
Right rostral middle frontal gyrus surface area	0.9654
Left lateral ventricles volume	0.9628
Left middle temporal gyrus surface area	0.9569
Left precuneus cortical thickness	0.9446
Left medial orbitofrontal cortical thickness	0.9379
**Gaussian Process Regression**	
**Feature**	**SHAP value**
Total Intracranial Volume	4.9332
Left Superior frontal gyrus Cortical thickness	3.2885
Right precuneus surface area	3.2639
Right middle temporal gyrus surface area	2.9548
Left thalamus volume	2.6361
Left hippocampus volume	2.1959
Left pericalcarine gyrus surface area	2.1729
Left precuneus surface area	2.1036
Right rostral middle frontal gyrus surface area	2.0488
Left superior frontal gyrus surface area	1.8995
Left lateral orbitofrontal gyrus surface area	1.8229
Right inferior parietal lobule cortical thickness	1.7784

Right insula surface area	1.7700
Right putamen volume	1.6108
Left superior temporal gyrus surface area	1.5758
Left precuneus cortical thickness	1.5754
Right superior frontal gyrus surface area	1.5263
Right hippocampus volume	1.5207
Right superior parietal lobule cortical thickness	1.4359
Right pars orbitalis surface area	1.3637
**Gradient Boosting Machine**	
**Feature**	**SHAP value**
Right lateral ventricles volume	1.1698
Left lateral ventricles volume	1.0374
Left thalamus volume	0.8868
Right pars triangularis cortical thickness	0.6585
Right putamen volume	0.6178
Left pars opercularis cortical thickness	0.6138
Right superior frontal gyrus cortical thickness	0.6012
Left precentral gyrus cortical thickness	0.4913
Left nucleus accumbens volume	0.4817
Left superior frontal gyrus cortical thickness	0.4548
Right nucleus accumbens volume	0.4176
Right entorhinal cortex surface area	0.4167
Left rostral anterior cingulate cortex cortical thickness	0.3975
Right pars opercularis cortical thickness	0.3707
Left pars triangularis cortical thickness	0.3424
Right thalamus volume	0.3240
Right paracentral cortical thickness	0.3120
Right middle temporal gyrus surface area	0.3112
Right rostral middle frontal gyrus cortical thickness	0.3086
Right insula cortical thickness	0.3031

Supplementary Table S7. The algorithm performance based on the structural features from the HCP individuals entered in the model for model performance in the training data (n = 890) and prediction performance in the hold-out test data (n = 223).

Algorithm	Model performance				Prediction performance			
	R	MAE	Weighted MAE	RMSE	R	MAE	Weighted MAE	RMSE
**Lasso**	0.4921	2.6444	0.1763	3.2266	0.4258	2.7565	0.1838	3.3425
**Lasso LAR**	0.4921	2.6444	0.1763	3.2266	0.4258	2.7565	0.1838	3.3425
**SVR**	0.4515	2.6981	0.1799	3.3156	0.4268	2.7756	0.1850	3.3378
**LAR**	0.4723	2.6933	0.1796	3.2779	0.4124	2.7896	0.1860	3.3964
**Elastic Net**	0.4714	2.6737	0.1782	3.2688	0.4199	2.7919	0.1861	3.3469
**Bayesian Ridge**	0.4712	2.6745	0.1783	3.2697	0.4182	2.7927	0.1862	3.3514
**Ridge**	0.4698	2.6797	0.1786	3.2706	0.4255	2.7941	0.1863	3.3353
**ARD**	0.4973	2.6373	0.1758	3.2408	0.3991	2.8251	0.1883	3.4551
**Random Forest**	0.4245	2.7785	0.1852	3.3648	0.4131	2.8304	0.1887	3.3757
**PAR**	0.4563	2.7231	0.1815	3.3643	0.4010	2.8322	0.1888	3.4451
**CatBoost**	0.4282	2.7631	0.1842	3.3465	0.4069	2.8328	0.1889	3.3682
**RVR**	0.4498	2.7148	0.1810	3.3407	0.4021	2.8371	0.1891	3.4017
**LightGBM**	0.4273	2.7457	0.1830	3.3473	0.4016	2.8418	0.1895	3.3782
**GBM**	0.4458	2.7149	0.1810	3.3086	0.4000	2.8437	0.1896	3.3805
**kNN**	0.3768	2.8367	0.1891	3.4310	0.3801	2.8591	0.1906	3.4166
**AdaBoost**	0.3982	2.8003	0.1867	3.3975	0.4188	2.8595	0.1906	3.3701
**Extra Trees**	0.4224	2.7738	0.1849	3.3750	0.4197	2.8674	0.1912	3.3793
**XGBoost**	0.4201	2.7726	0.1848	3.3709	0.3859	2.8771	0.1918	3.4145
**Kernel Ridge**	0.4417	2.7495	0.1833	3.4199	0.3878	2.8775	0.1918	3.5100
**GPR**	0.4735	2.7199	0.1813	3.3656	0.3689	2.9420	0.1961	3.6260
**MLP**	0.4744	2.7216	0.1814	3.3665	0.3675	2.9450	0.1963	3.6365
**OMP**	0.4790	2.6927	0.1795	3.3220	0.3590	2.9457	0.1964	3.6112
**LR**	0.4736	2.7244	0.1816	3.3695	0.3679	2.9474	0.1965	3.6353
**Huber**	0.4705	2.7366	0.1824	3.3859	0.3674	2.9484	0.1966	3.6416
**Theil-Sen**	0.4663	2.7544	0.1836	3.4066	0.3398	2.9724	0.1982	3.7220
**RANSAC**	0.4553	2.8094	0.1873	3.4962	0.3627	3.0015	0.2001	3.7149
**Decision Tree**	0.1694	3.0653	0.2044	3.6947	0.1122	3.1206	0.2080	3.6980

Supplementary Table S8. The algorithm performance based on the structural features from the Cam-CAN individuals entered in the model for model performance in the training data (n = 500) and prediction performance in the hold-out test data (n = 101).

Algorithm	Model performance				Prediction performance			
	R	MAE	Weighted MAE	RMSE	R	MAE	Weighted MAE	RMSE
**Lasso LAR**	0.8952	6.6767	0.0954	8.2589	0.8589	7.0830	0.1012	8.9581
**ARD**	0.8992	6.5372	0.0934	8.1039	0.8585	7.1040	0.1015	8.9490
**Lasso**	0.8943	6.6898	0.0956	8.3192	0.8567	7.1757	0.1025	9.0092
**Elastic Net**	0.8960	6.6632	0.0952	8.2187	0.8548	7.1816	0.1026	9.0706
**Huber**	0.8938	6.7060	0.0958	8.2835	0.8455	7.4663	0.1067	9.3166
**Bayesian Ridge**	0.8927	6.7691	0.0967	8.3097	0.8445	7.4698	0.1067	9.3459
**RVR**	0.8824	6.9355	0.0991	8.6688	0.8378	7.5311	0.1076	9.5367
**PAR**	0.8877	6.9834	0.0998	8.6292	0.8395	7.5762	0.1082	9.4878
**Ridge**	0.8906	6.8230	0.0975	8.4733	0.8432	7.5865	0.1084	9.3765
**OMP**	0.8827	7.0357	0.1005	8.7920	0.8437	7.6179	0.1088	9.3874
**GPR**	0.8839	7.0175	0.1003	8.8069	0.8377	7.7190	0.1103	9.5526
**LR**	0.8826	7.0582	0.1008	8.8650	0.8366	7.7432	0.1106	9.5853
**MLP**	0.8831	7.0570	0.1008	8.8420	0.8364	7.7450	0.1106	9.5878
**SVR**	0.8887	6.8523	0.0979	8.4799	0.8309	7.7551	0.1108	9.7319
**RANSAC**	0.8789	7.2202	0.1031	9.0337	0.8282	7.8652	0.1124	9.8203
**Theil-Sen**	0.8791	7.1771	0.1025	9.0597	0.8366	7.8698	0.1124	9.6104
**GBM**	0.8681	7.3435	0.1049	9.1562	0.8368	7.9222	0.1132	9.6339
**CatBoost**	0.8667	7.3767	0.1054	9.2270	0.8230	8.1285	0.1161	10.0429
**XGBoost**	0.8552	7.5686	0.1081	9.5496	0.8167	8.3920	0.1199	10.2965
**LightGBM**	0.8646	7.1822	0.1026	9.2233	0.8040	8.4686	0.1210	10.4704
**Kernel Ridge**	0.876	7.2091	0.1030	8.9549	0.7022	8.6938	0.1242	14.1861
**Extra Trees**	0.8565	7.7800	0.1111	9.6758	0.8050	8.8377	0.1263	10.4629
**Random Forest**	0.8410	8.0043	0.1143	10.0129	0.7955	8.9883	0.1284	10.6854
**AdaBoost**	0.8405	8.0458	0.1149	10.0575	0.7725	9.4055	0.1344	11.1983
**LAR**	0.8378	8.3740	0.1196	10.3008	0.7577	9.5307	0.1362	11.4450
**kNN**	0.8234	8.7403	0.1249	10.7922	0.7709	9.6734	0.1382	11.5064
**Decision Tree**	0.7259	9.7473	0.1392	12.7333	0.6430	10.5017	0.1500	13.9457

Supplementary Table S9. The algorithm performance based on the structural features from the IXI individuals entered in the model for model performance in the training data (n = 453) and prediction performance in the hold-out test data (n = 114).

Algorithm	Model performance				Prediction performance			
	R	MAE	Weighted MAE	RMSE	R	MAE	Weighted MAE	RMSE
**ARD**	0.8268	7.4790	0.1133	9.3023	0.7998	8.0453	0.1219	9.7586
**Lasso LAR**	0.8290	7.4126	0.1123	9.2080	0.7981	8.0473	0.1219	9.8065
**Lasso**	0.8290	7.4129	0.1123	9.2085	0.7981	8.0477	0.1219	9.8067
**MLP**	0.7939	8.1039	0.1228	10.364	0.7779	8.0675	0.1222	10.3325
**PAR**	0.8171	7.8135	0.1184	9.7111	0.7902	8.2368	0.1248	9.9684
**XGBoost**	0.8160	7.7096	0.1168	9.5314	0.7918	8.2664	0.1252	9.9961
**Bayesian Ridge**	0.8308	7.4376	0.1127	9.2152	0.7945	8.2785	0.1254	9.8881
**GBM**	0.8161	7.5873	0.1150	9.5333	0.7818	8.3159	0.1260	10.2182
**Elastic Net**	0.8343	7.3865	0.1119	9.1223	0.7947	8.3217	0.1261	9.9199
**SVR**	0.8303	7.5350	0.1142	9.2671	0.7904	8.3845	0.1270	9.9973
**Ridge**	0.8329	7.4285	0.1126	9.1804	0.7934	8.3912	0.1271	9.9448
**GPR**	0.7866	8.4452	0.1280	10.5033	0.7719	8.3925	0.1272	10.4667
**LAR**	0.8132	7.7176	0.1169	9.6041	0.7837	8.4347	0.1278	10.1105
**LR**	0.7832	8.5274	0.1292	10.6074	0.7692	8.4450	0.1280	10.5417
**Huber**	0.7966	8.1947	0.1242	10.2374	0.7704	8.5157	0.1290	10.5225
**CatBoost**	0.8299	7.6574	0.1160	9.2785	0.7918	8.6085	0.1304	10.2038
**Theil-Sen**	0.7862	8.4097	0.1274	10.5505	0.7534	8.6277	0.1307	10.9854
**RVR**	0.8322	7.4849	0.1134	9.2289	0.7766	8.6291	0.1307	10.2432
**OMP**	0.8029	7.9480	0.1204	9.8892	0.7603	8.8267	0.1337	10.5987
**LightGBM**	0.8196	7.6084	0.1153	9.492	0.7475	8.8588	0.1342	10.7975
**Extra Trees**	0.8257	7.7683	0.1177	9.5285	0.7876	8.9449	0.1355	10.4976
**Random Forest**	0.8118	7.9223	0.1200	9.7984	0.7679	8.9912	0.1362	10.7648
**Kernel Ridge**	0.8316	7.5138	0.1138	9.1818	0.7230	9.0415	0.1370	11.2986
**RANSAC**	0.7772	8.655	0.1311	10.931	0.7384	9.1059	0.1380	11.4204
**AdaBoost**	0.8211	7.7603	0.1176	9.4691	0.7402	9.2366	0.1399	11.0331
**kNN**	0.7769	8.3113	0.1259	10.4212	0.7027	9.2521	0.1402	11.7032
**Decision Tree**	0.7066	9.3118	0.1411	12.2431	0.6315	9.8640	0.1495	13.2255

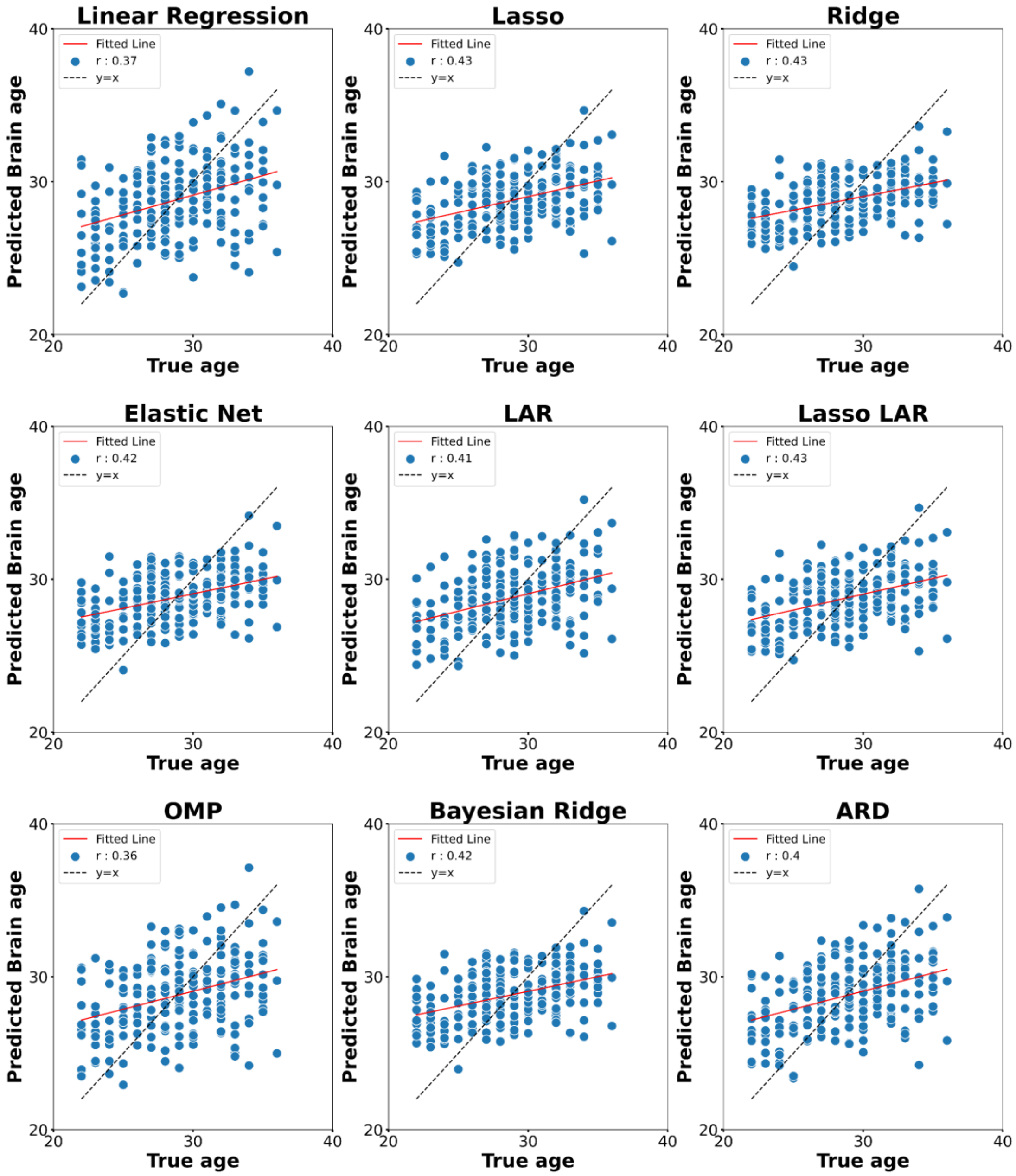

Linear Regression
Fitted Line
r : 0.37
y=x
Lasso
Fitted Line
r : 0.43
y=x
Ridge
Fitted Line
r : 0.43
y=x
Elastic Net
Fitted Line
r : 0.42
y=x
LAR
Fitted Line
r : 0.41
y=x
Lasso LAR
Fitted Line
r : 0.43
y=x
OMP
Fitted Line
r : 0.36
y=x
Bayesian Ridge
Fitted Line
r : 0.42
y=x
ARD
Fitted Line
r : 0.4
y=x
Predicted Brain age
True age
20
30
40

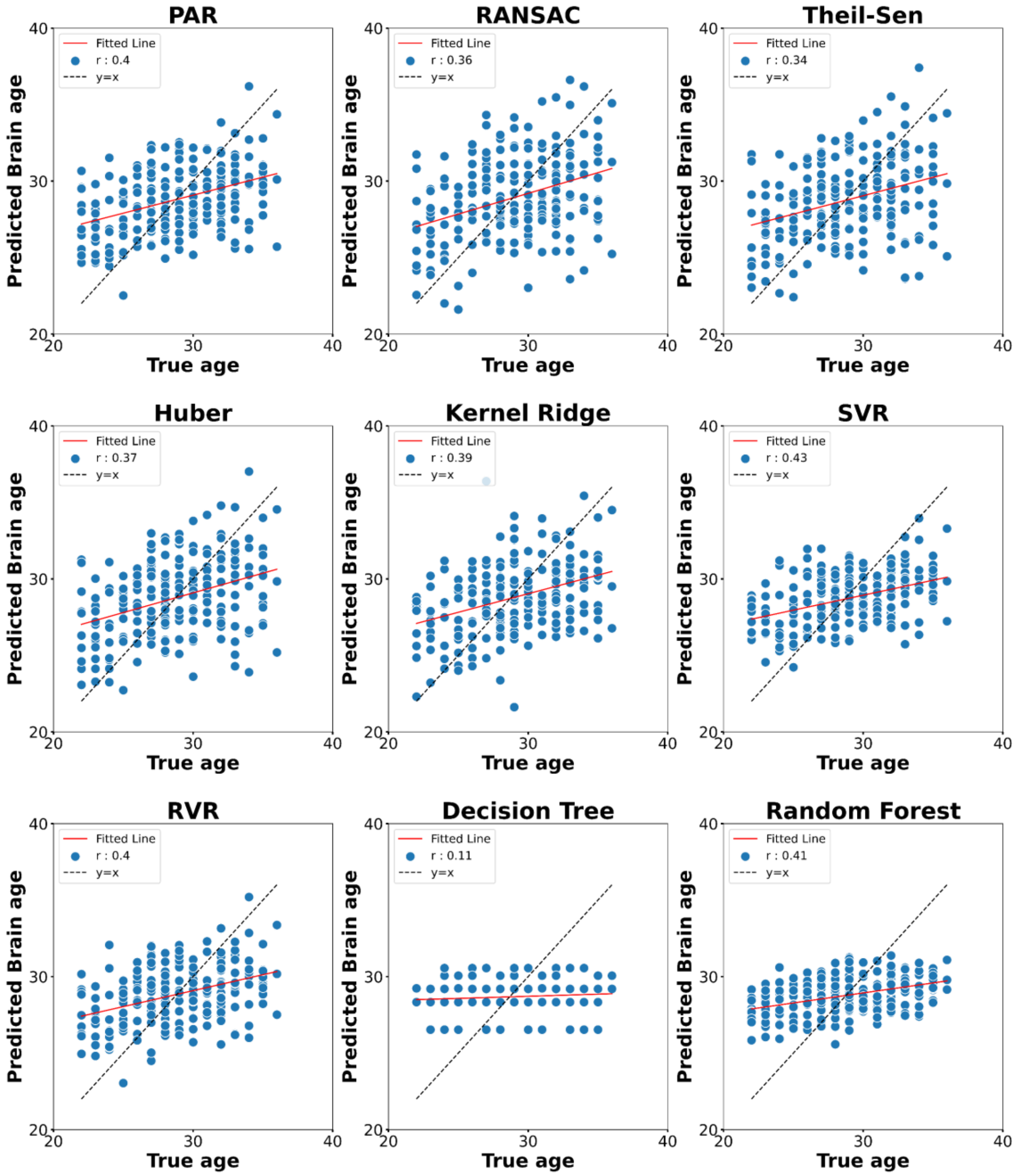
PAR
RANSAC
Theil-Sen
Huber
Kernel Ridge
SVR
RVR
Decision Tree
Random Forest
Fitted Line
r : 0.4
r : 0.36
r : 0.34
r : 0.37
r : 0.39
r : 0.43
r : 0.4
r : 0.11
r : 0.41
y=x
Predicted Brain age
True age
20
30
40

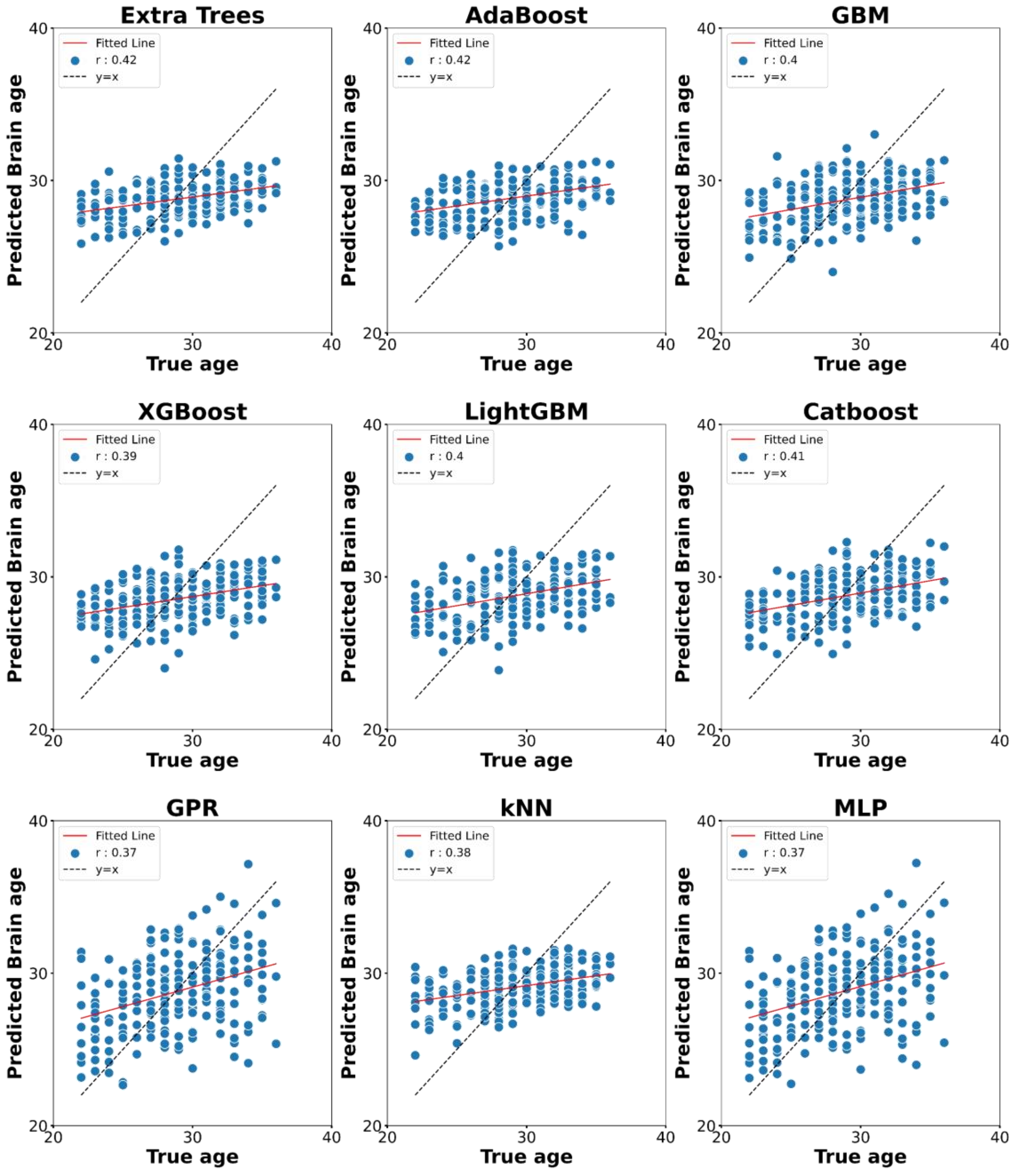

Supplementary Figure S2. Scatter plots showing pairwise correlations between chronological age and predicted brain age across 27 algorithms for the HCP cohort. Pearson correlations are shown for each association. Lasso = Least Absolute Shrinkage and Selection Operator Regression; Lasso LAR = Lasso Least Angle Regression; SVR = Support Vector Regression; LAR= Least Angle Regression; Elastic Net = Elastic-Net Regression; Bayesian Ridge = Bayesian Ridge Regression; Ridge = Ridge Regression; ARD = Automatic Relevance

Determination; Random Forest = Random Forest Regression; PAR = Passive Aggressive Regression; CatBoost = Category Boosting Regression; RVR = Relevance Vector Regression; LightGBM= Light Gradient Boosting Machine; GBM = Gradient Boosting Machine; kNN = K-Nearest Neighbors; AdaBoost = Adaptive Boosting Regression; Extra Trees = Extra Trees Regression; XGBoost = Extreme Gradient Boosting; Kernel Ridge = Kernel Ridge Regression; GPR = Gaussian Processes Regression; MLP = Multi-layer Perceptron Regression; OMP = Orthogonal Matching Pursuit; Linear Regression = Linear Regression; Huber = Huber Regression; Theil-Sen = Theil-Sen Regression; RANSAC = Random Sample Consensus; Decision Tree = Decision Tree Regression.

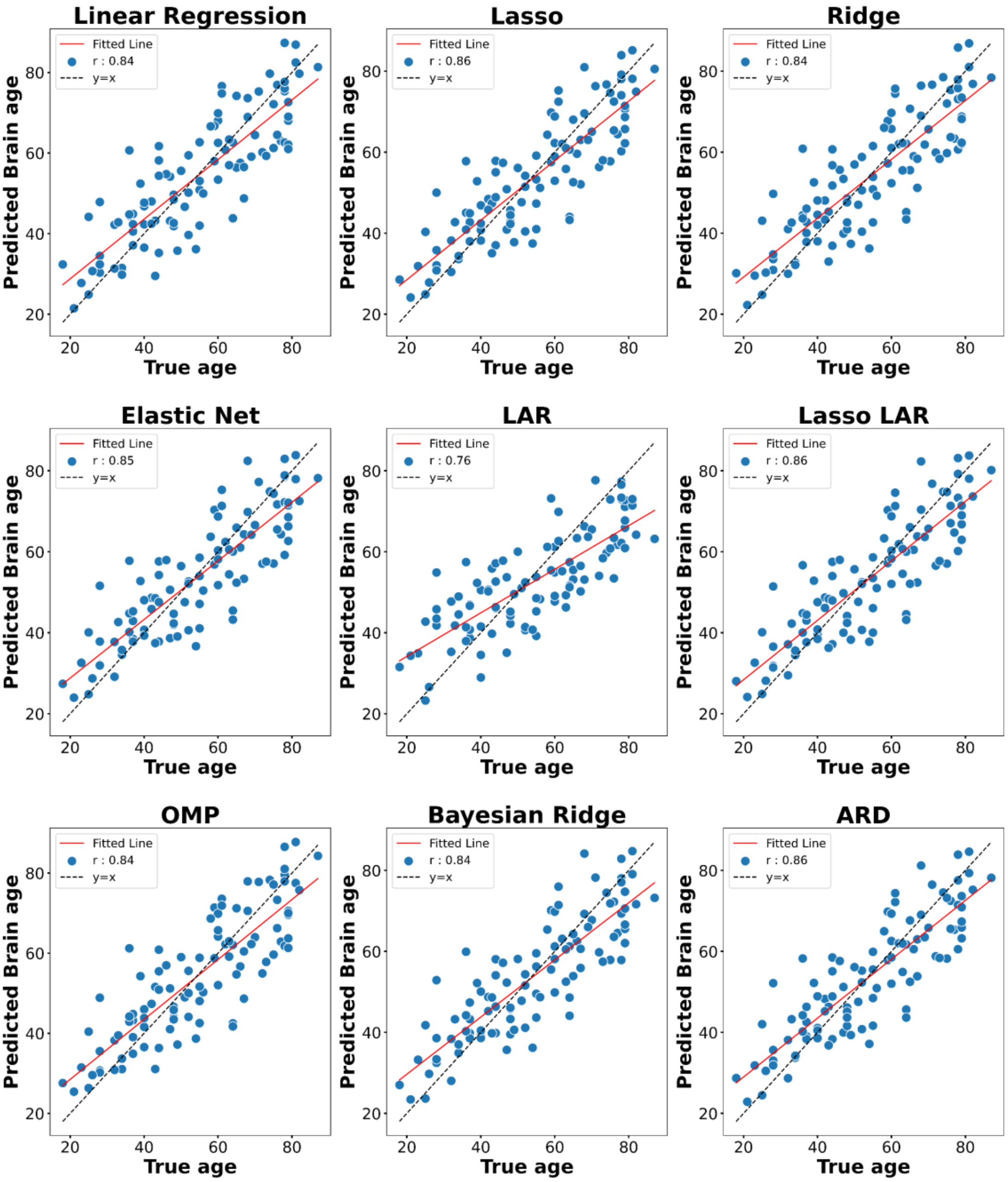
Linear Regression
Fitted Line
r : 0.84
y=x
Lasso
Fitted Line
r : 0.86
y=x
Ridge
Fitted Line
r : 0.84
y=x
Elastic Net
Fitted Line
r : 0.85
y=x
LAR
Fitted Line
r : 0.76
y=x
Lasso LAR
Fitted Line
r : 0.86
y=x
OMP
Fitted Line
r : 0.84
y=x
Bayesian Ridge
Fitted Line
r : 0.84
y=x
ARD
Fitted Line
r : 0.86
y=x
Predicted Brain age
True age
20
40
60
80

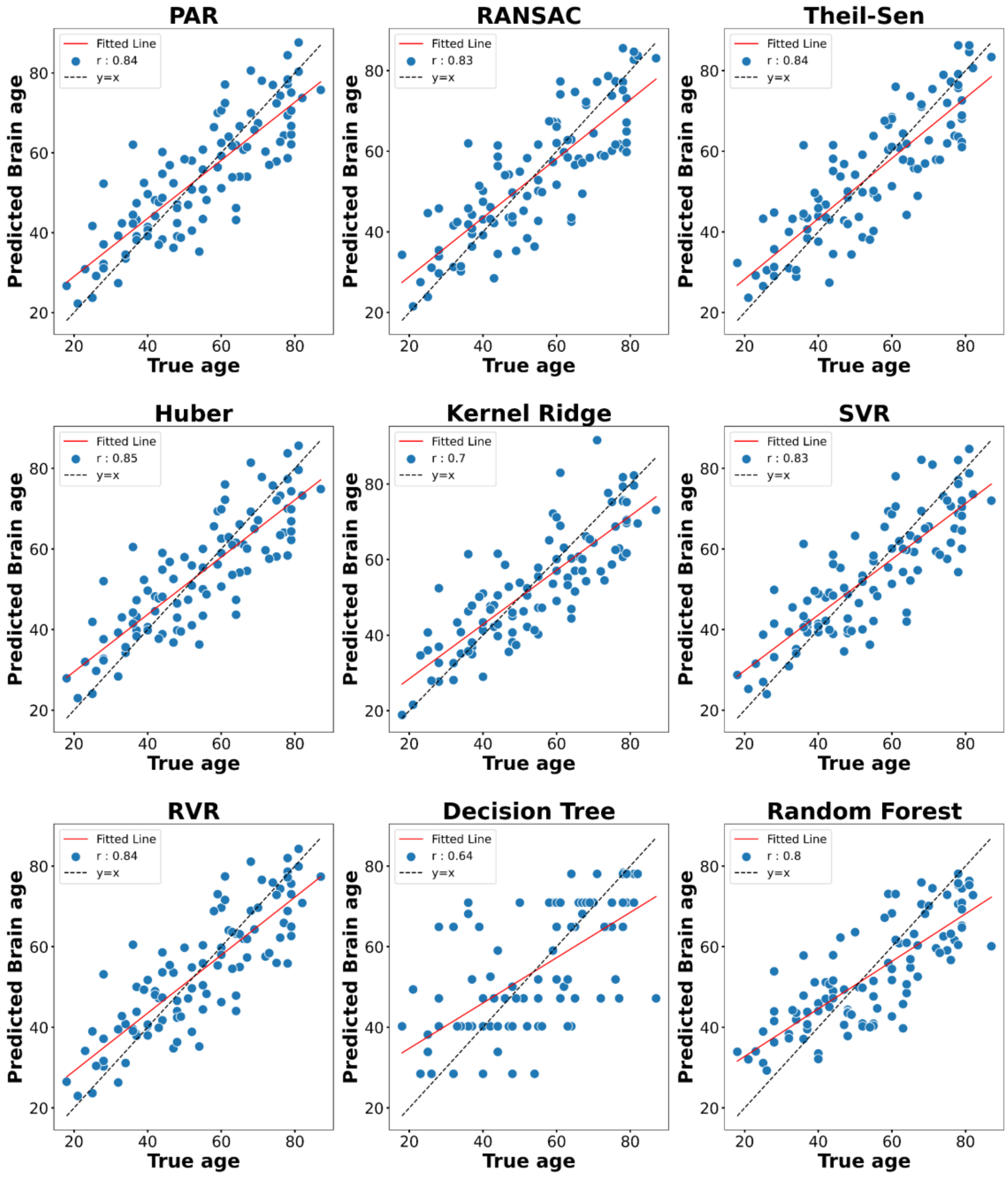

PAR
Fitted Line
r : 0.84
y=x
Predicted Brain age
True age
RANSAC
r : 0.83
Theil-Sen
r : 0.84
Huber
r : 0.85
Kernel Ridge
r : 0.7
SVR
r : 0.83
RVR
r : 0.84
Decision Tree
r : 0.64
Random Forest
r : 0.8

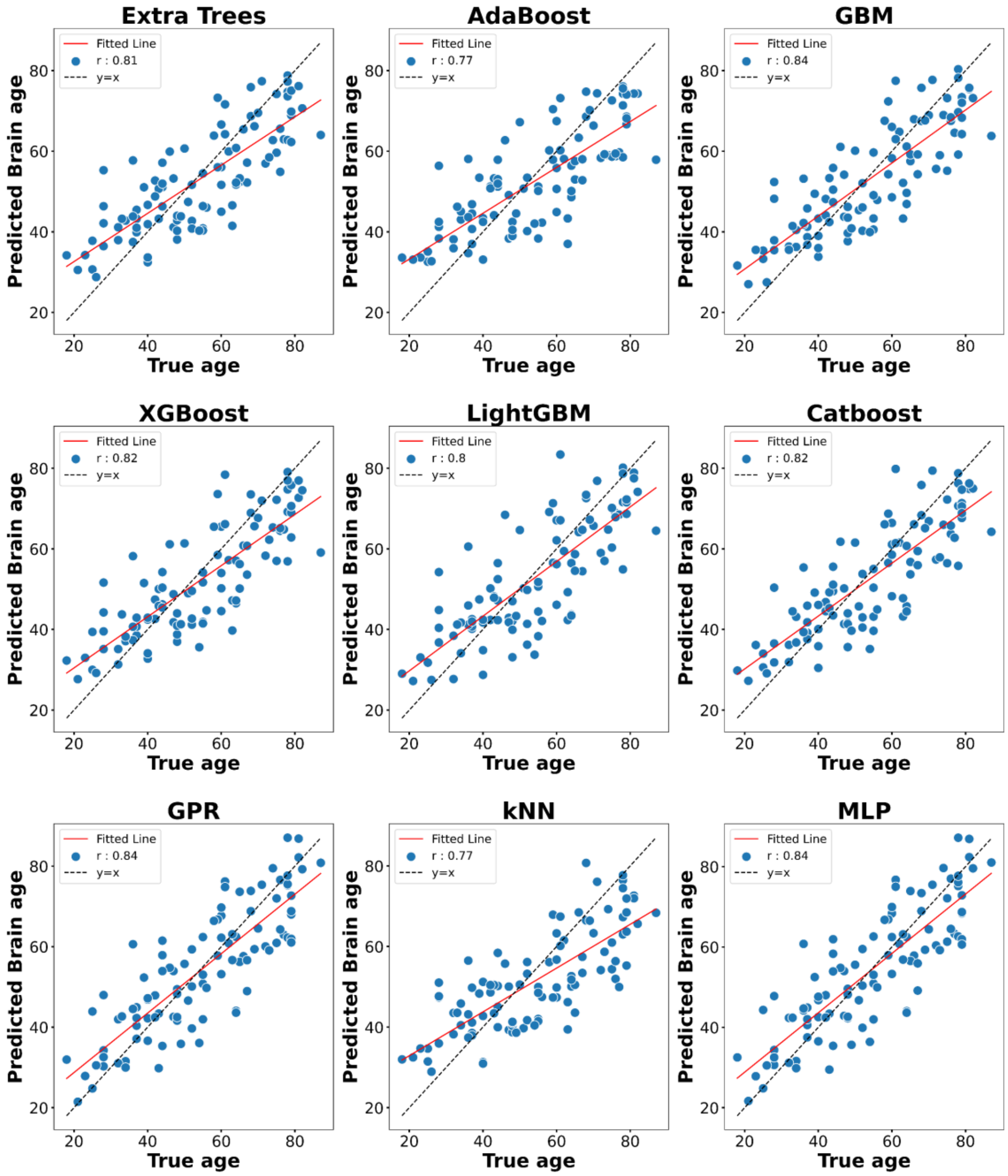

Supplementary Figure S3. Scatter plots showing pairwise correlations between chronological age and predicted brain age across 27 algorithms for the Cam-CAN cohort. Pearson correlations are shown for each association. Lasso = Least Absolute Shrinkage and Selection Operator Regression; Lasso LAR = Lasso Least Angle Regression; SVR = Support Vector Regression; LAR= Least Angle Regression; Elastic Net = Elastic-Net Regression; Bayesian Ridge = Bayesian Ridge Regression; Ridge = Ridge Regression; ARD = Automatic

Relevance Determination; Random Forest = Random Forest Regression; PAR = Passive Aggressive Regression; CatBoost = Category Boosting Regression; RVR = Relevance Vector Regression; LightGBM= Light Gradient Boosting Machine; GBM = Gradient Boosting Machine; kNN = K-Nearest Neighbors; AdaBoost = Adaptive Boosting Regression; Extra Trees = Extra Trees Regression; XGBoost = Extreme Gradient Boosting; Kernel Ridge = Kernel Ridge Regression; GPR = Gaussian Processes Regression; MLP = Multi-layer Perceptron Regression; OMP = Orthogonal Matching Pursuit; Linear Regression = Linear Regression; Huber = Huber Regression; Theil-Sen = Theil-Sen Regression; RANSAC = Random Sample Consensus; Decision Tree = Decision Tree Regression.

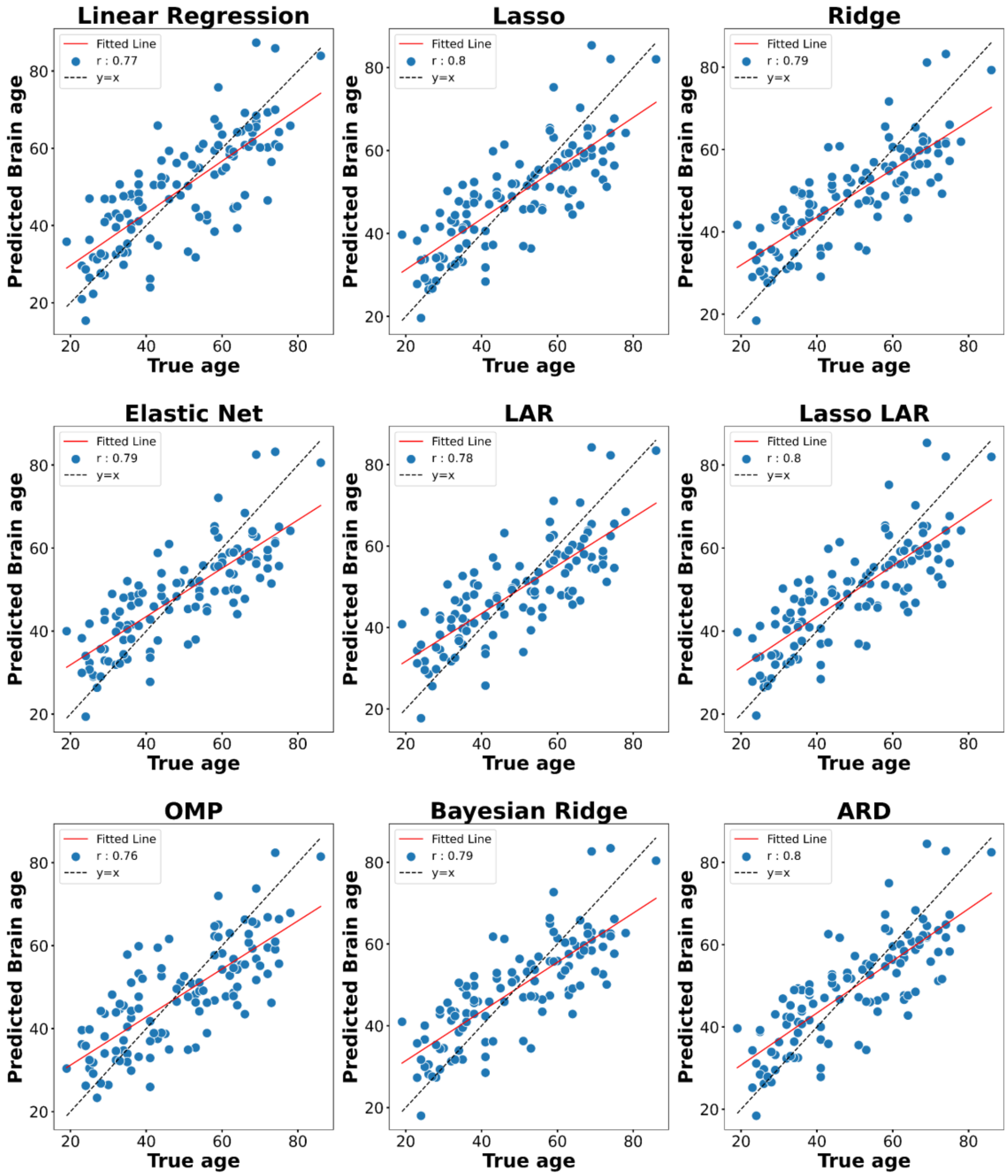
Linear Regression
Fitted Line
r : 0.77
y=x
Lasso
Fitted Line
r : 0.8
y=x
Ridge
Fitted Line
r : 0.79
y=x
Elastic Net
Fitted Line
r : 0.79
y=x
LAR
Fitted Line
r : 0.78
y=x
Lasso LAR
Fitted Line
r : 0.8
y=x
OMP
Fitted Line
r : 0.76
y=x
Bayesian Ridge
Fitted Line
r : 0.79
y=x
ARD
Fitted Line
r : 0.8
y=x
Predicted Brain age
True age
20
40
60
80

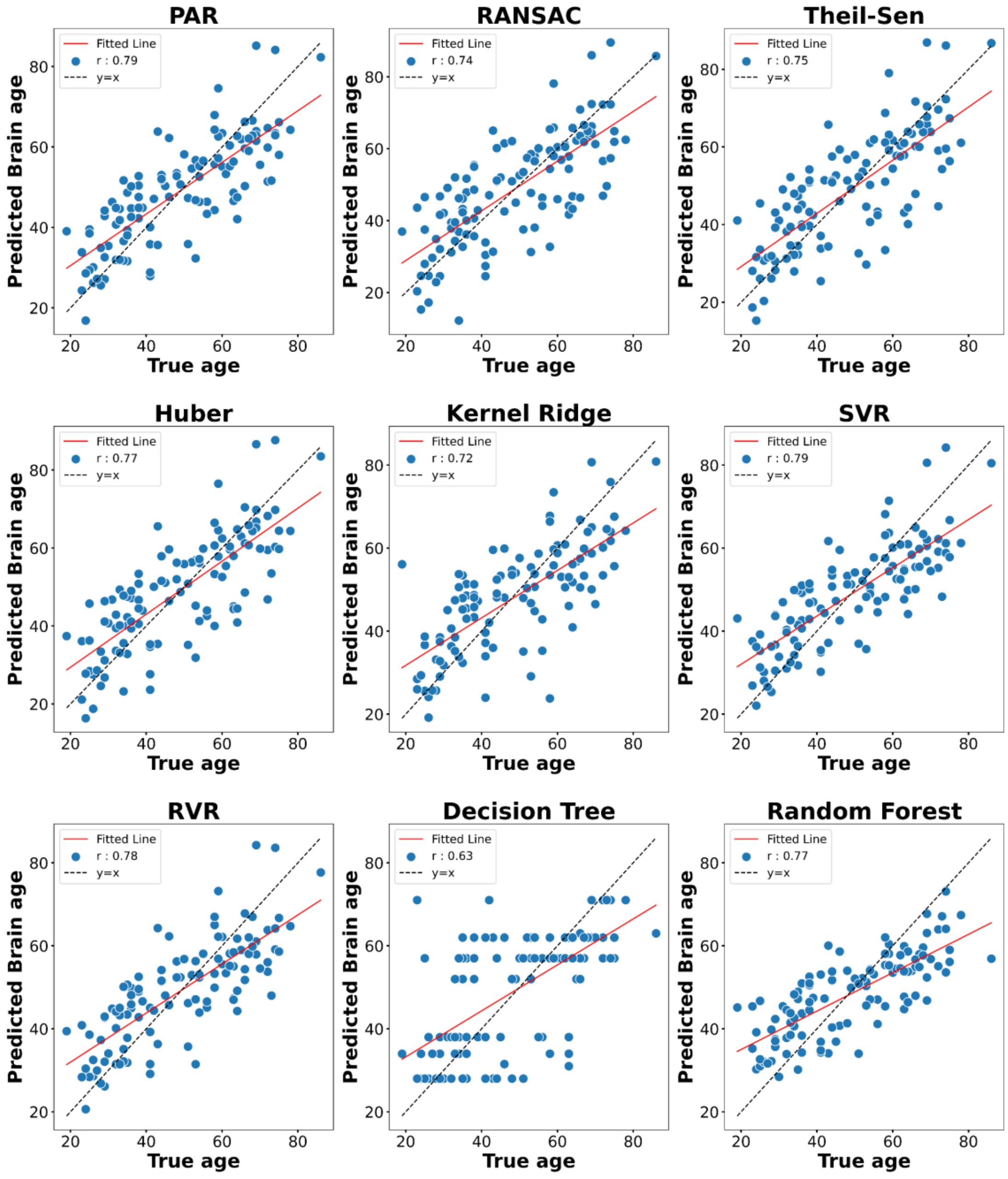

PAR
Fitted Line
r : 0.79
y=x
RANSAC
r : 0.74
Theil-Sen
r : 0.75
Huber
r : 0.77
Kernel Ridge
r : 0.72
SVR
r : 0.79
RVR
r : 0.78
Decision Tree
r : 0.63
Random Forest
r : 0.77
Predicted Brain age
True age

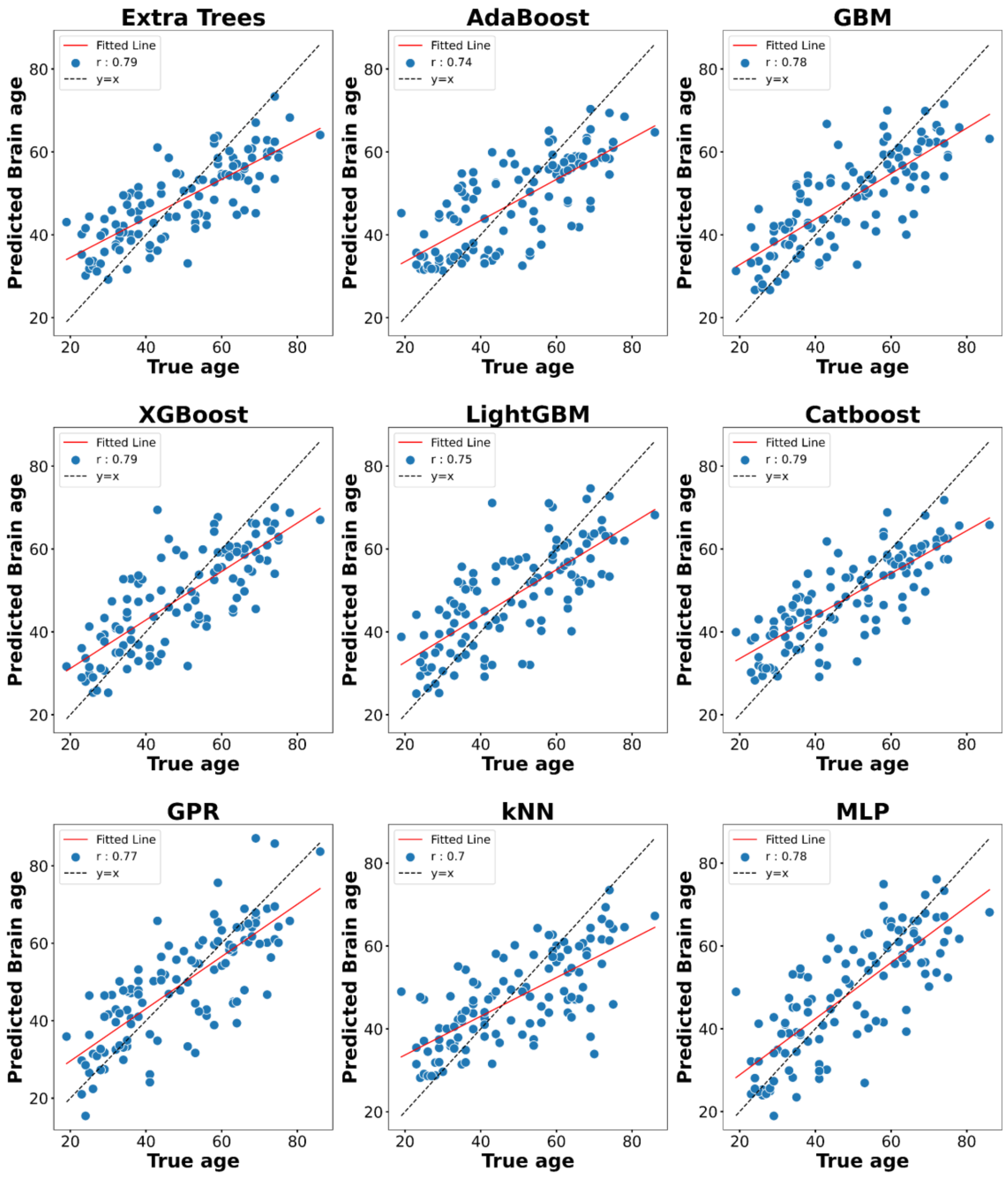

Supplementary Figure S4. Scatter plots showing pairwise correlations between chronological age and predicted brain age across 27 algorithms for the IXI cohort. Pearson correlations are shown for each association. Lasso = Least Absolute Shrinkage and Selection Operator Regression; Lasso LAR = Lasso Least Angle Regression; SVR = Support Vector Regression; LAR= Least Angle Regression; Elastic Net = Elastic-Net Regression; Bayesian Ridge = Bayesian Ridge Regression; Ridge = Ridge Regression; ARD = Automatic Relevance

Determination; Random Forest = Random Forest Regression; PAR = Passive Aggressive Regression; CatBoost = Category Boosting Regression; RVR = Relevance Vector Regression; LightGBM= Light Gradient Boosting Machine; GBM = Gradient Boosting Regression; kNN = K-Nearest Neighbors; AdaBoost = Adaptive Boosting Regression; Extra Trees = Extra Trees Regression; XGBoost = Extreme Gradient Boosting; Kernel Ridge = Kernel Ridge Regression; GPR = Gaussian Processes Regression; MLP = Multi-layer Perceptron Regression; OMP = Orthogonal Matching Pursuit; Linear Regression = Linear Regression; Huber = Huber Regression; Theil-Sen = Theil-Sen Regression; RANSAC = Random Sample Consensus; Decision Tree = Decision Tree Regression.

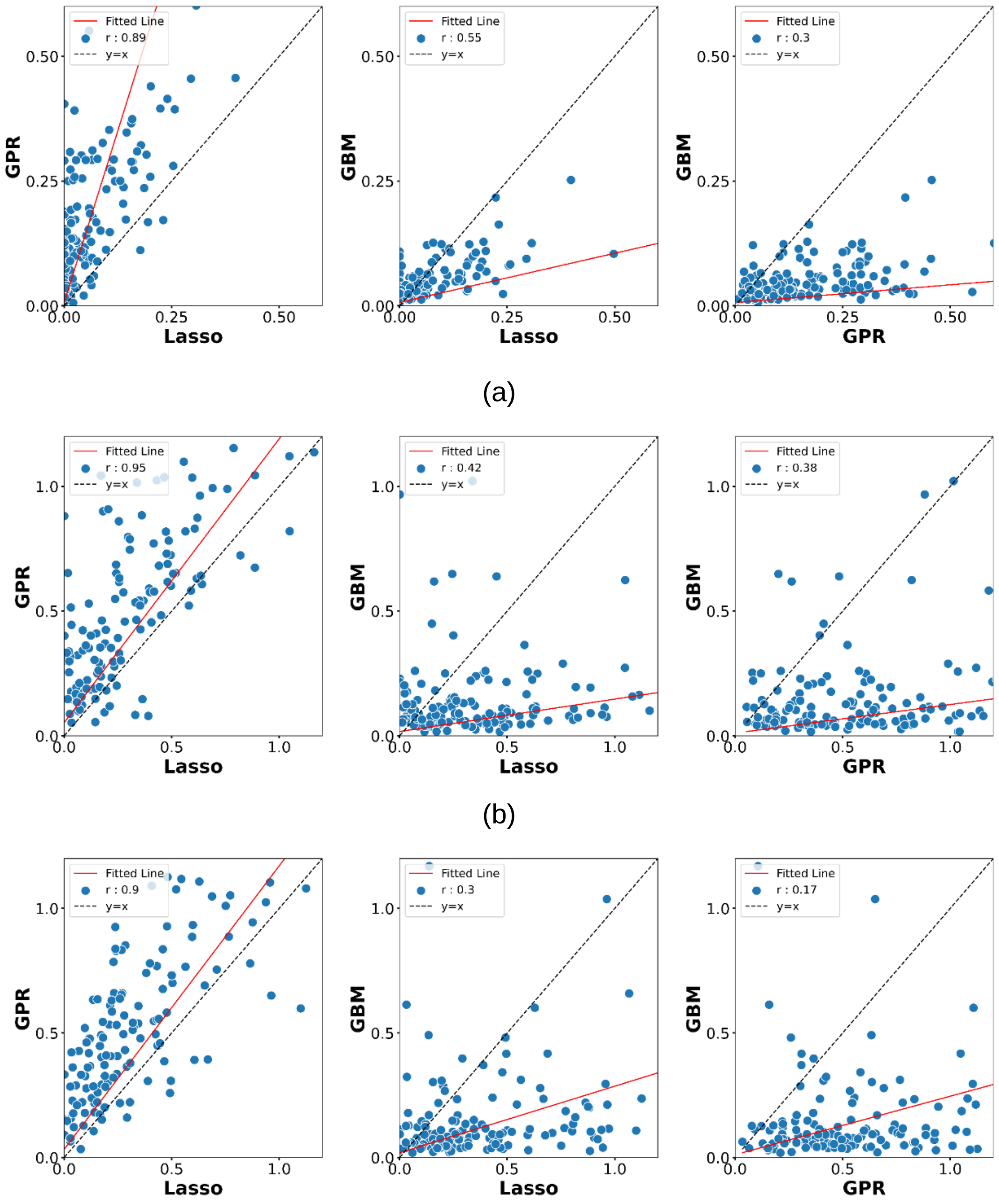

(c)

Supplementary Figure S5. Scatter plots showing correlations of mean absolute SHAP values (feature importance) between models (Lasso, GPR, and GBM) for the (a) HCP, (b) Cam-CAN, and IXI cohorts. Pearson correlations are shown for each association. Lasso = Least Absolute Shrinkage and Selection Operator Regression; GPR = Gaussian Processes Regression; GBM = Gradient Boosting Machine.

The effects of feature selection on regression performance

Feature selection was performed based on the SHAP values (i.e., feature importance) to select an optimal subset of features. We trained each model (i.e., Lasso, Gaussian process regression, and gradient boosting machine) with the top n (from 1 to 153 with a step of 1) feature(s) in the training set and estimated the regression performance on the test with the trained model. For each dataset, we present the results below for the regression performance using top n (from 1to 153 with a step size of 1) features(s) by each algorithm. We report the MAE values averaged across all cross-validation results.

In the HCP, using the top 38 features, an average MAE was 2.71 in the test set, lower than 2.75, corresponding to the lowest MAE obtained by all 153 features with the Lasso. Gaussian process regression with the top 67 features achieved an average MAE of 2.81 in the test set, which is lower than 2.94, equal to the lowest MAE obtained by all 153 features. Gradient boosting machine with the top 52 features achieved an average MAE value of 2.75 in the test set, lower than 2.84, corresponding to the lowest MAE obtained by all 153 features. In the Cam-CAN, using the top 121 features, an average MAE was 7.11 in the test set, lower than 7.17, corresponding to the lowest MAE obtained by all 153 features with the Lasso. Gaussian process regression with the top 27 features achieved an average MAE of 7.18 in the test set, which is lower than 7.71, equal to the lowest MAE obtained by all 153 features. Gradient boosting machine with the top 75 features achieved an average MAE value of 7.37 in the test set, lower than 7.92 corresponding to the lowest MAE obtained by all 153 features. In the IXI, using the top 78 features, an average MAE was 8.04 in the test set, equal to the lowest MAE obtained by all 153 features with the Lasso. Gaussian process regression with the top 92 features achieved an average MAE of 8.13 in the test set, which is lower than 8.39, equal to the lowest MAE obtained by all 153 features. Gradient boosting machine with the top 31 features achieved an average MAE value of 7.97 in the test set, lower than 8.31, corresponding to the lowest MAE obtained by all 153 features.

We selected the most important features based on the SHAP value and eliminated features with low SHAP values to select an optimal subset of features. Then we investigated the effects of feature selections on algorithm performance. Our results demonstrate that feature selection improves the performance of each algorithm in brain age prediction

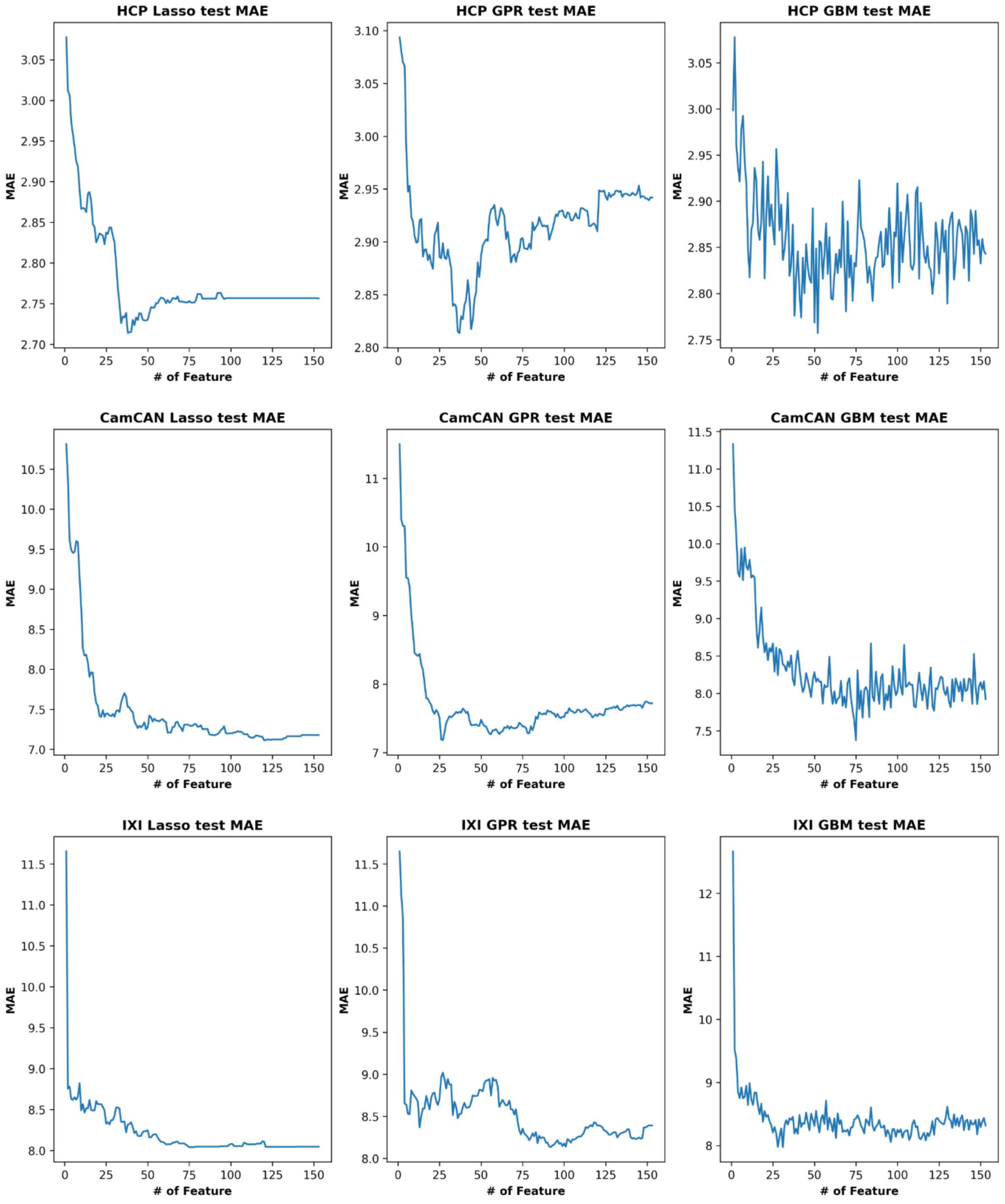

Supplementary Figure S6. Average accuracies (mean absolute error, MAE) of different feature dimensionality in the test sets for each algorithm (Lasso, Gaussian process regression, and gradient boosting machine) in the HCP (top), Cam-CAN (middle), and IXI (bottom).
